# Supplementary material for: Synapsin shields a lipid monolayer from interactions with vesicles and associated structural rearrangements
Source: Biophys J. 2025 Dec 4;125(12):3118–27. doi: 10.1016/j.bpj.2025.12.004 (PMC13507188; doi:10.1016/j.bpj.2025.12.004)
Supplement: Supplementary file 1 [file mmc1.pdf]

Biophysical Journal, Volume *125*

Supplemental information

Synapsin shields a lipid monolayer from interactions with vesicles and associated structural rearrangements

Hendrik Bruns, Titus Czajka, Charlotte Neuhaus, Maciej Jankowski, Diego Pontoni, Christian Hoffmann, Dragomir Milovanovic, and Tim Salditt

Supporting Material

Synapsin shields a lipid monolayer which is contact with synaptic vesicles or small lipid vesicles from structural rearrangement

Hendrik Bruns[1,‖], Titus Czajka[1,‖], Charlotte Neuhaus[1], Maciej Jankowski[3], Diego Pontoni[3], Christian Hoffmann[2], Dragomir Milovanovic[2], and Tim Salditt[1,*]

[1]Institute for X-ray physics, Friedrich-Hund-Platz 1, Göttingen, 37077, Germany
[2]Laboratory of Molecular Neuroscience, German Center for Neurodegenerative Diseases (DZNE), 10117 Berlin, Germany
[3] ESRF - European Synchrotron Radiation Facility, 71 avenue des Martyrs, 3800 Grenoble, France
[‖]These authors contributed equally
[*]Correspondence: tsaldit@gwdg.de

A GRAZING INCIDENCE DIFFRACTION: LATTICE PARAMETERS AND PEAK FITTING

The GID data presented in Fig. 2,3(e) is the result of fitting the $\{11\}$ and $\{02\}$ peaks in the 2d plane shown in Fig. S1. The two-dimensional crystalline packing of the lipid chains is described by the lattice vectors a and b of a rectangular lattice. The GID peaks with inplane component of the scattering vector q_{hk} (Miller indices) are related to the lattice vectors as (1)

$$q_{hk} = \frac{1}{d_{hk}} = \frac{\sqrt{h^2b^2+k^2a^2}}{ab}\,. \tag{1}$$

From q_{02}, we can directly infer the lattice parameter b

$$q_{02} = \frac{1}{d_{02}} = \frac{\sqrt{0^2b^2+2^2a^2}}{ab} = \frac{2}{b} \quad \Rightarrow \quad b = 2d_{02}\,. \tag{2}$$

This relation for b can then be inserted in the expression for q_{11} in Eq.(1)

$$q_{11} = \frac{1}{d_{11}} = \frac{\sqrt{1^2b^2+1^2a^2}}{ab} \tag{3}$$

$$\Rightarrow \quad d_{11} = \frac{ab}{\sqrt{b^2+a^2}} \overset{(2)}{=} \frac{a2d_{02}}{\sqrt{(2d_{02})^2+a^2}} = \frac{2d_{02}}{\sqrt{\left(\frac{2d_{02}}{a}\right)^2+1}} \tag{4}$$

$$\Rightarrow \quad \sqrt{\left(\frac{2d_{02}}{a}\right)^2+1} = \frac{2d_{02}}{d_{11}} \quad \Rightarrow \quad \frac{(2d_{02})^2}{a^2} = \frac{(2d_{02})^2}{d_{11}^2} - 1 \quad \Rightarrow \quad a^2 = \frac{(2d_{02})^2}{\frac{(2d_{02})^2}{d_{11}^2}-1} \tag{5}$$

$$\Rightarrow \quad a = \frac{2d_{02}}{\sqrt{\frac{(2d_{02})^2}{d_{11}^2}-1}} = \frac{1}{\sqrt{\frac{1}{d_{11}^2}-\frac{1}{(2d_{02})^2}}}\,. \tag{6}$$

From the lattice vectors a and b, one obtains the area per chain A and tilt angle τ, presented in Fig. 2,3,5,6. The corresponding 2d measurements in the $q_{x,y}$ and q_z plane are shown in Fig.S1, S2, S3. The dark lines indicate the fit results for the three relevant prak positions $q_{xy,11}$, $q_{z,11}$, and $q_{xy,02}$. The gray lines are added as a visual reference. The peak center is determined by projecting the intensity onto the respective axis and subsequently fitted to a Gaussian. The width is used to determine the error.

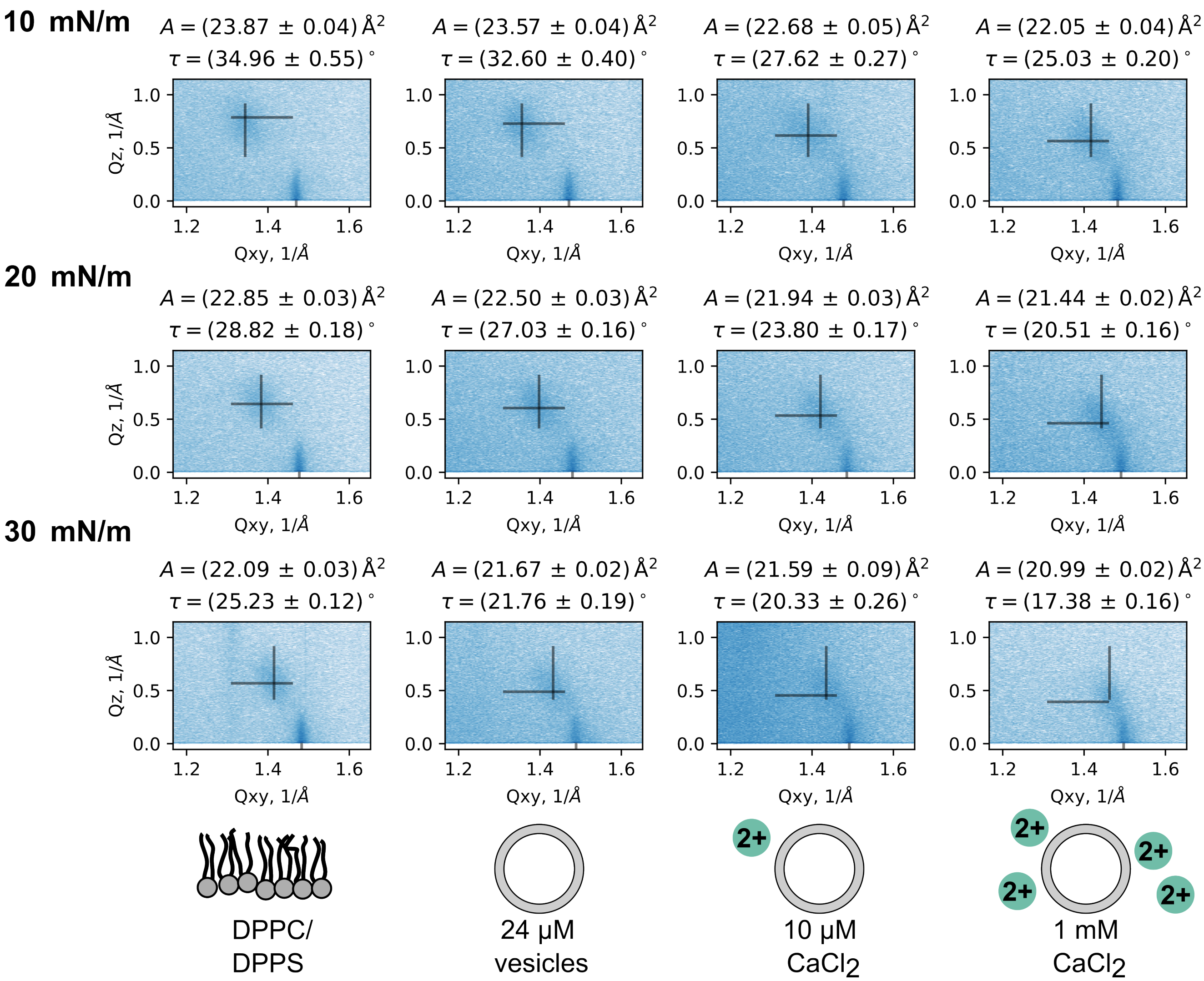

Figure S1: GID data collected at a constant pressure p, by scanning the linear 1d detector along angle 2θ. The linear 1d detector is aligned along the interface normal. Two peaks are observed; the gray lines indicate the fitted peak positions. From the peak positions (q_z^{11} q_{xy}^{11} q_{xy}^{02}) the area per chain A and the tilt angle τ is computed (see subpanel titles). The data corresponds to the A results presented in Fig.3(e).

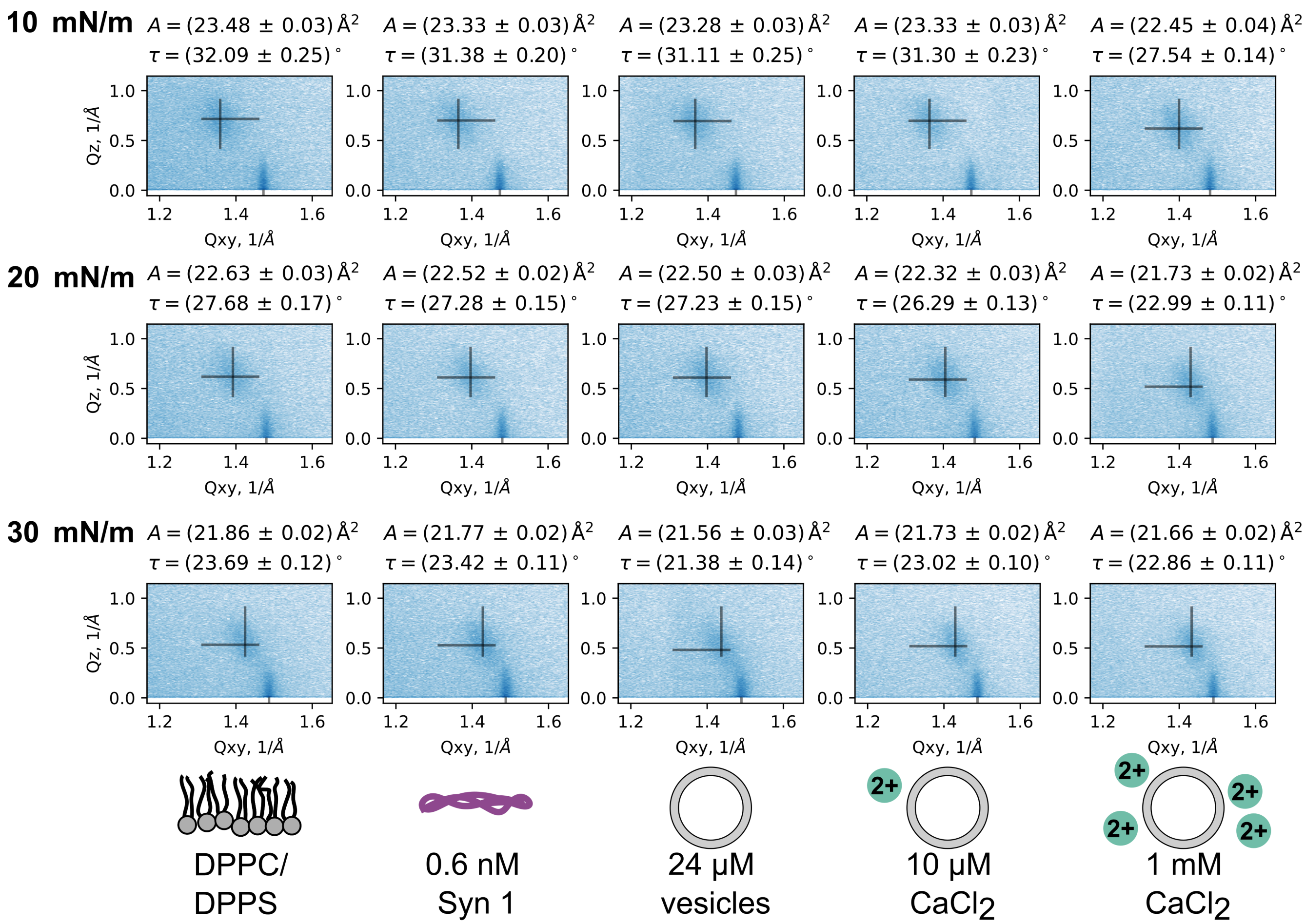

Figure S2: GID data corresponding to A values presented in Fig.2(e); else same as in Fig. above.

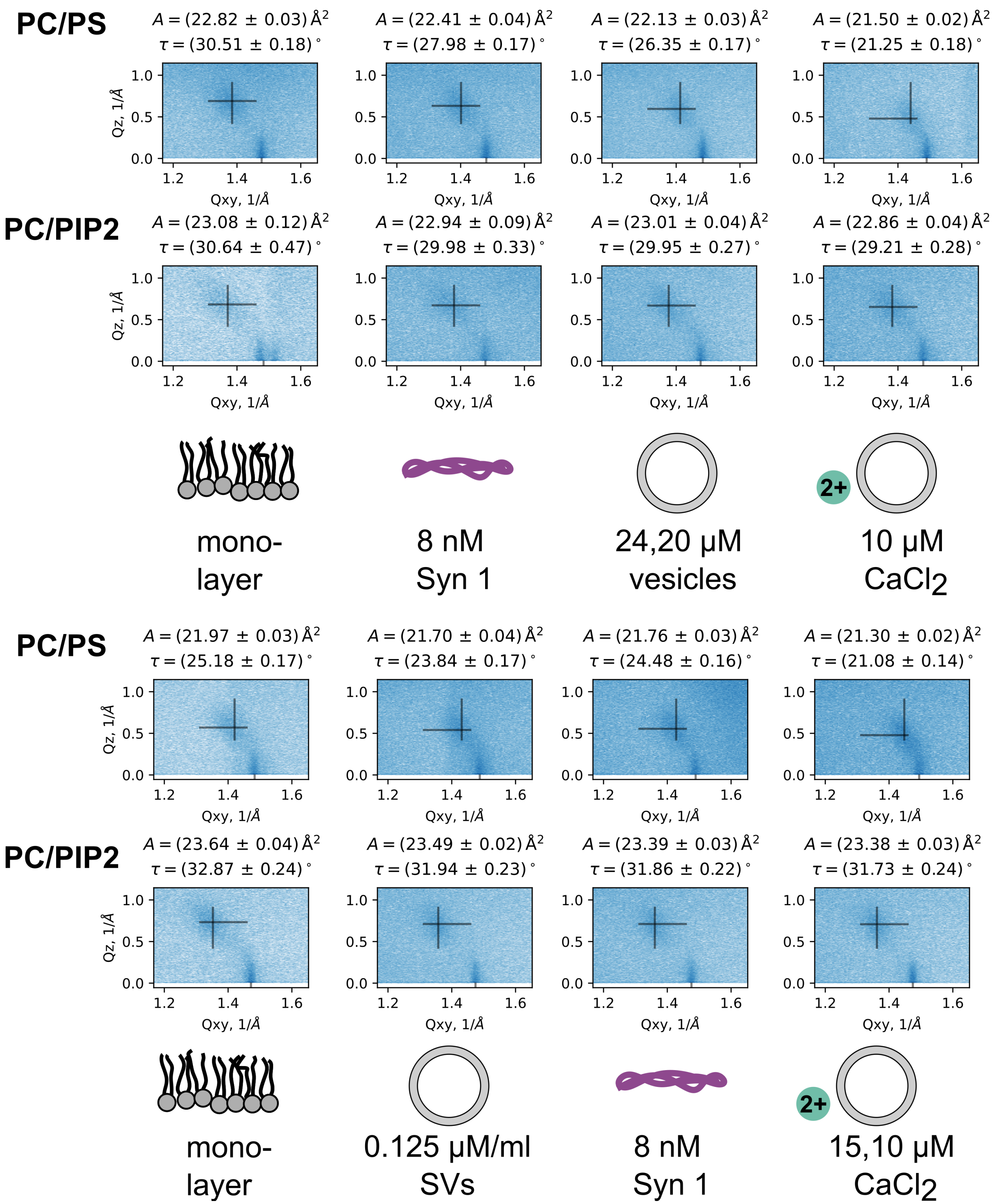

Figure S3: GID data from the small trough data, at constant area and approximate monolayer pressure of 20 mN/m. The 2d plots correspond to the data presented in Fig. 5,6.

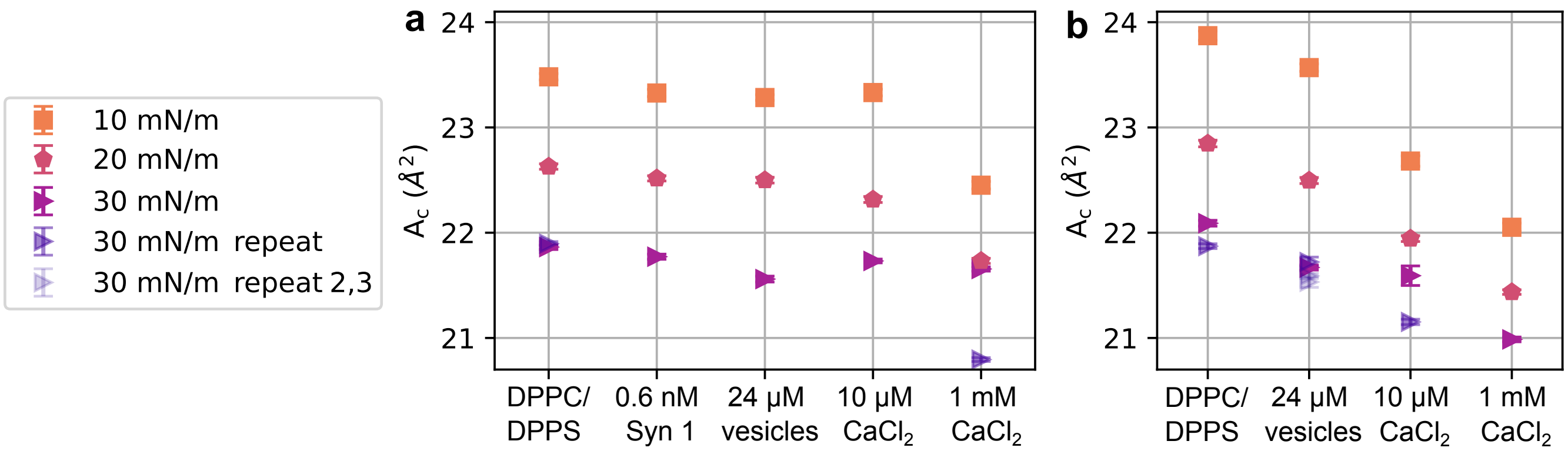

Figure S4: GID data obtained on the large trough, as presented in Fig. 2,3(e). Repeat measurements are shown at constant monolayer pressure of 30 mN/m. The 2d plots correspond to the data presented in Fig.S1 and S2

Repredicibility In order to check for reproducibility of the states of the individual monolayers in Fig. 2,3(e) repeated measurements where performed at a surface pressure 30 mN/m and are shown in purple in Fig. S4. The monolayer repeat measurements are acquired after going to the lower pressures showing a similar area per chain A_c. Fig. S4(b) shows three repeated measurements at 30 mN/m, following the acquisition of the two lower pressures. These three measurements show a slow decay of A_c which can be attributed to the reordering of lipids finding the optimal packing at this pressure. This could also explain the greater variations in A_c at 30 mN/m as the injection process varied in duration. Accordingly, the lipids were kept at 30 mN/m for a varying duration, while the measurement script for the lower film pressures consistently adjusted the trought to the desired pressure, and then waited for 5 min before starting the measurement. In addition, we note that the initial measurement of the 30 mN/m monolayer after $CaCl_2$ injection deviates from the repeated measurement (see Fig. S4 10 mM $CaCl_2$ (a) and 10 μM $CaCl_2$ (b)). This is indicative of either a delayed interaction or a required lower membrane surface pressure Π for the interaction.

B X-RAY REFLECTIVITY ANALYSIS

Since the XRR fits had a tendency towards overfitting with a three-box model, the modelling of a separate protein layer had to be given up. It was found, that the information that distinguished different sets of parameters from each other was contained most heavily at $q > 0.6\,\text{nm}^{-1}$, where the X-ray data was too close to the noise level to yield definitive results. A particular property of the three-box models was that they required the space between monolayer and subphase to have a density below that of the subphase, which was deemed to be an unphysical solution.

To reduce the complexity of the model, given the low information content available for higher spacial frequencies (high q), a two box model was chosen (as illustrated in Fig. S6). This reduced the number of parameters and thus led to less variation in the fit results for different sets of initial parameters, but still did not yield a single best fit result. To achieve this, we further constrained the model by setting the the interfacial width σ equal for all three interfaces. Furthermore, we set an upper limit for the tail density $\rho_t < (0.93 \cdot \rho_s)$.

The fitting procedure itself comprised a least-squares minimisation algorithm which was fed different sets of parameters. As criterion for the goodness of fit, the adjusted coefficient of determination R^2_{adj} was used. To test the validity of the calculated best least-squares fit from a certain set of input parameters, we chose different parameters based on the concept of a Metropolis algorithm: The set of parameters that is an input to each fit is taken as the initial probability distribution and the resulting value of R^2_{adj} corresponds to the energy of the system in the original algorithm. The algorithm then iterates through multiple sets of parameters by proposing a new parameter configuration based on the old configuration, but now each individual parameter is shifted by an amount $\pm\Delta p_i < p_i$ chosen from a normal distribution centred around p_i. This new set of parameters is accepted when $(R_i/R_{i+1})^n > U[0;1]$, where $U[0;1]$ denotes a uniform random number in the interval between 0 and 1. The goal of the algorithm is to obtain a large variety of different fits that are on the other hand not sampling too many configurations where the least-squares fit algorithm fails. We obtained good results by choosing $n = 4$ and $\Delta p_i = \mathcal{N}(p_i, \Delta * p_i)$ where Δ was varied from 0.5 to 0.002.

Even though the least-squares minimisation is not a Markovian process, and the Metropolis algorithm is hence not strictly applicable here, we nonetheless found it to be a reasonable way to generate a multitude of different fit parameters and output configurations. The different configurations then allowed us to assess whether the models yielded different fit results of similar

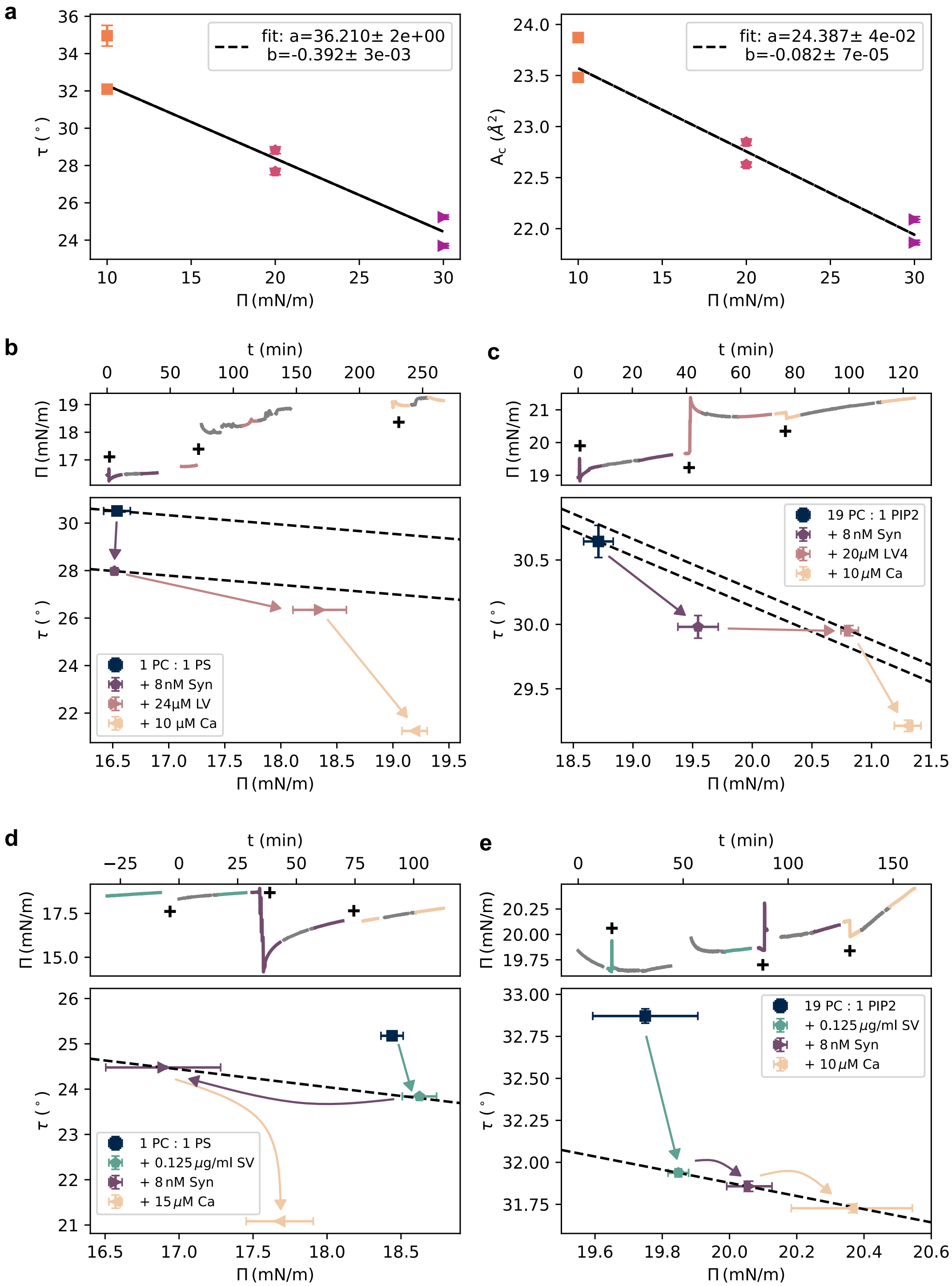

Figure S5: (a) Monolayer pressure dependence of the chain tilt angle τ and area per chain A_c, along with linear fits. These fits are then used as reference isotherms in (b-e) (τ) and in Fig. 5,6 (A_c). (b-e) Tilt angle τ in response to injections of Syn1, lipid vesicle, synaptic vesicles and $CaCl_2$, corresponding to Fig. 5,6 (main manuscript). The response in τ is similar to A_c.

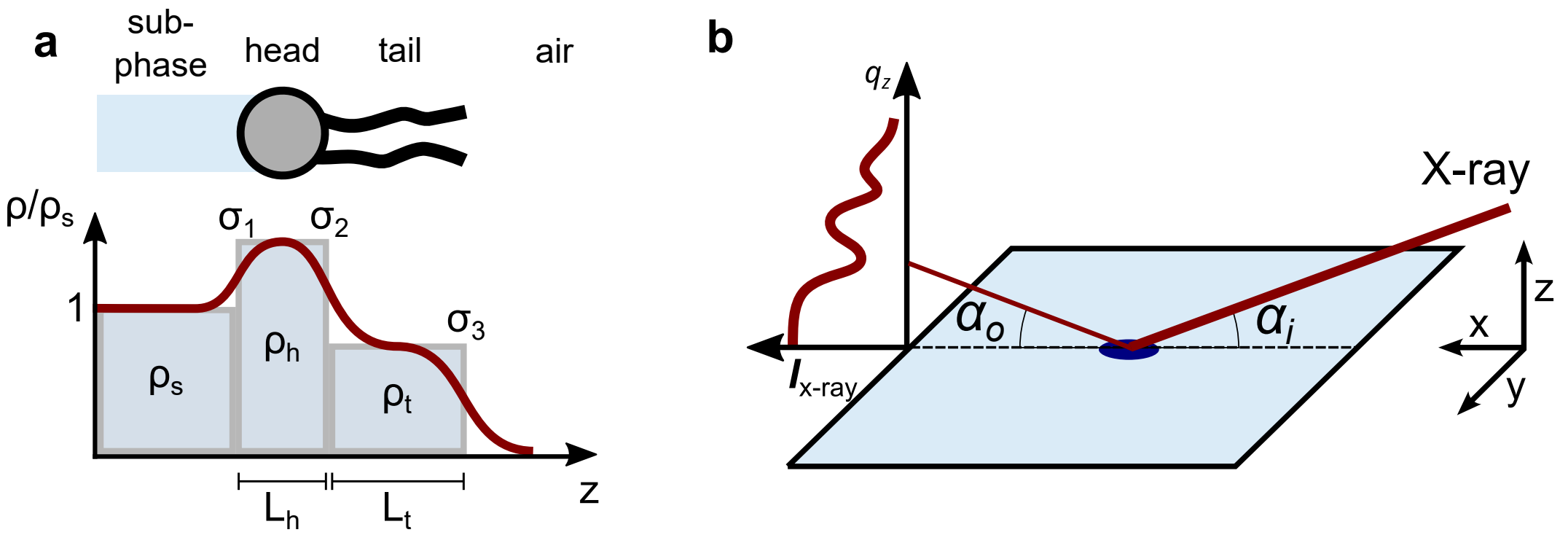

Figure S6: (a) XRR two box density model for the lipid head and tail group, illustrating the parameterisation of the density profile ρ. These parameters are varied to fit the reflectivity curves recorded as sketched in (b).

R^2_{adj} or rather a single global optimum.

REFERENCES

1. Marsh, D., 2012. Lateral order in gel, subgel and crystalline phases of lipid membranes: Wide-angle X-ray scattering. *Chemistry and physics of lipids* 165:59–76.
