# Supplementary material for: Synapsin shields a lipid monolayer from interactions with vesicles and associated structural rearrangements
Source: Biophys J. 2025 Dec 4;125(12):3118–27. doi: 10.1016/j.bpj.2025.12.004 (PMC13507188; doi:10.1016/j.bpj.2025.12.004)
Supplement: Supplementary file 2 [file mmc2.pdf]

Biophysical Journal
Article

Biophysical Society

# Synapsin shields a lipid monolayer from interactions with vesicles and associated structural rearrangements

Hendrik Bruns,[1] Titus Czajka,[1] Charlotte Neuhaus,[1] Maciej Jankowski,[4] Diego Pontoni,[4] Christian Hoffmann,[2,3] Dragomir Milovanovic,[2,3] and Tim Salditt[1,*]

[1]Institute for X-ray Physics, Friedrich-Hund-Platz 1, Göttingen, Germany; [2]Institute of Biochemistry, Charité-Universitätsmedizin Berlin, Corporate Member of Freie Universität Berlin, Humboldt-Universität Berlin, and Berlin Institute of Health, Berlin, Germany; [3]Laboratory of Molecular Neuroscience, German Center for Neurodegenerative Diseases (DZNE), Berlin, Germany; and [4]ESRF – European Synchrotron Radiation Facility, Grenoble, France

ABSTRACT Synapsins are the proteins responsible for recruiting synaptic vesicles into the synaptic vesicle cluster. As one of the most abundant synaptic proteins, synapsins are well known to directly interact with the synaptic vesicles, but they can also interact with planar membranes, notably the synaptic membrane, at least indirectly by tethering vesicles to the active zone. In order to contribute to a quantitative understanding of how interactions with synapsin affect the structure of a membrane already before neurotransmission, we use a minimal in vitro model of a lipid monolayer in contact with a subphase containing synapsin 1 and vesicles. We then probe the interface structure by x-ray reflectivity and grazing incidence diffraction at controlled surface pressure and monitor changes in lipid chain packing in the presence and absence of synapsin. In the absence of synapsin, injection of vesicles into the subphase below the film causes a pronounced reduction in lipid chain spacing, which further decreases by addition of calcium. This effect was muted when the film was first incubated with synapsin before injection of vesicles. We interpret this as a protective function of synapsin suppressing perturbation of the synaptic membrane, which could result in unwanted spontaneous fusion.

SIGNIFICANCE We monitor the structural response of a lipid monolayer after injection of vesicles into the subphase of the Langmuir film balance, with and without prior incubation with synapsin. We observe that synapsin shields the interface from a strong response and structural rearrangements associated with vesicle interaction. This "protective role" of synapsin may possibly help to prevent unwanted spontaneous fusion of vesicles with the synaptic membrane.

## INTRODUCTION

Synapsins are known as highly abundant cytosolic proteins in the presynaptic terminals of neurons (1). They are tightly associated with synaptic vesicles (SVs) (2), the trafficking organelles responsible for transport and release of neurotransmitters into the synaptic cleft, after fusion with the synaptic membrane (3). The main role of synapsins is to regulate vesicle trafficking, clustering, and neurotransmitter release, in particular the formation of the synaptic vesicle cluster (SVC), an essential organelle of the synapse (4). The synapsin-rich SVC stores neurotransmitter-loaded SVs and conditions them for fusion with the synaptic membrane and subsequent neurotransmitter release during synaptic activity. It also controls the local concentrations of numerous exo- and endocytosis cofactors (5) and can be considered as a membraneless compartment, formed by liquid-liquid phase separation (LLPS), which in turn is largely driven by synapsin (6). Loosely speaking, synapsins organize the SVC, whereas the SVC itself can be regarded as a major spatial organizing unit of the presynapse. For the underlying interactions, the intrinsically disordered region of synapsin seems to play a critical role (7,8). The interaction is also known to depend on membrane curvature (favoring small vesicles) and on the presence of anionic lipids, in particular phosphatidylserines (9,10). Additionally, synapsin was shown to be highly surface active (11). Finally, in the N-terminal region

*Submitted July 31, 2025, and accepted for publication December 1, 2025.*
*Correspondence: tsaldit@gwdg.de
Hendrik Bruns and Titus Czajka contributed equally to this work.
Editor: John Conboy.
https://doi.org/10.1016/j.bpj.2025.12.004
© 2025 The Author(s). Published by Elsevier Inc. on behalf of Biophysical Society.
This is an open access article under the CC BY license (http://creativecommons.org/licenses/by/4.0/).

(B-domain), synapsins can form an amphipathic $\alpha$-helix, which would allow partial insertion into membranes (10,11).

When addressing the basic interactions of synapsin with membranes, two very different targets for potential interaction have to be distinguished: the outer leaflet of the highly curved vesicular membrane and the essentially planar inner leaflet of the synaptic membrane. For both targets, important biophysical principles can be inferred from in vitro models (5). For the SVC, condensates of synapsin and vesicles represent such a minimal model. In fact, even in buffered solution, synapsin induces LLPS on its own, but it has also been shown to integrate both synaptic vesicles as well as lipid vesicles into condensates by interacting with the vesicular membranes (6,7). Phase behavior, morphology, and phase transitions of condensates (12), as well as the intervesicle distances (13), have been studied in bulk solutions of such minimal models. Langmuir monolayers with controlled lipid composition (14) have been used to target the interaction of SVs with planar lipid interfaces (15). To this end, SVs were injected into the subphase below the monolayer, and the interface structure was then probed by x-ray reflectivity (XRR) and grazing incidence diffraction (GID) at different $Ca^{2+}$ concentration (16). By recording the surface pressure increase at constant film area, after injection of vesicles into the subphase, exchange of lipids from vesicles to the monolayer could be evidenced.

Toward an understanding of the modulating role that synapsin has in these interactions of vesicles and lipid interfaces, we here extend the lipid monolayer approach and probe structural changes in the lipid film in the absence and presence of synapsin, followed by different subsequent steps of vesicle and $Ca^{2+}$ injection. Fig. 1 presents the experimental approach, where the presynaptic membrane is represented by the monolayer, and molecular changes of the film in response to synapsin, and vesicle interaction can be probed by diffraction. To this end, we use two different lipid monolayer compositions, three different monolayer pressures, synapsin, and different concentrations of $Ca^{2+}$, as well as SV purified from rat brain and/or small unilamellar lipid vesicles (LV) of controlled lipid composition. The results reported below are surprising and very clear: prior binding of synapsin shields the target membrane from structural changes when exposed to SVs or LVs or higher $Ca^{2+}$ levels. Contrarily, in the absence of synapsin the lipid chains change conformation when SVs and/or $Ca^{2+}$ are added. As we discuss at the end of this manuscript, synapsin may therefore also have a protective role, preventing unwanted strong interactions when SV fusion is not forced by SNARE proteins.

## MATERIALS AND METHODS

### General

Lipids for the monolayer and vesicles were purchased as powders from Avanti Polar Lipids (Alabaster, AL, USA). Lipid monolayers were made from 1,2-dipalmitoyl-sn-glycero-3-phosphocholine (DPPC), 1,2-dipalmitoyl-sn-glycero-3-phospho-L-serine (DPPS), and 1,2-dioleoyl-sn-glycero-3-phospho-(1′-myo-inositol-4′,5′-bisphosphate) ($PIP_2$). The monolayer composition was either a 1:1 mix of DPPC and DPPS or a 19:1 mix of DPPC and $PIP_2$ for all x-ray experiments (by molar fraction). LVs were prepared from 1,2-di-(9Z-octadecenoyl)-sn-glycero-3-phosphocholine (DOPC), 1,2-di-(9Z-octadecenoyl)-sn-glycero-3-phospho-L-serine (DOPS), 1,2-di-(9Z-octadecenoyl)-sn-glycero-3-phosphoethanolamine (DOPE), and cholesterol. All measurements were performed using Tris-buffered-saline (TBS) in the subphase and as a buffer for vesicles and the protein. The buffer was prepared by mixing 25 mM Tris-HCl, 150 mM NaCl, and 0.5 mM TCEP and adjusting to pH 7.4 with NaOH. The expression and purification of synapsin 1a protein was performed as previously described by Hoffmann et al. (17). Synaptic vesicles were purified according to the protocol given by Takamori et al. (18).

### Lipid vesicles

LVs were prepared from stock solutions (10 mg/mL, 2 $CHCl_3$:1 MeOH) to yield a lipid composition of 55 $\%_{mol}$ DOPC, 15 $\%_{mol}$ DOPE, 20 $\%_{mol}$ DOPS, and 10 $\%_{mol}$ cholesterol. Solvent was evaporated under a stream of $N_2$ and subsequently dried under vacuum for >4 h. Lipid films were resuspended with TBS buffer and vortexed until all lipids were dissolved. Vesicles were formed by 10 freeze-thaw cycles in liquid nitrogen and a 37°C water bath. Finally, the vesicle solution was extruded 21 times through a 50-nm polycarbonate membrane (Mini Extruder, Avanti Polar Lipids, AL, USA) to form small unilamellar vesicles comparable in size to synaptic vesicles. All lipid vesicle concentrations are given as preextrusion lipid concentrations.

### Langmuir monolayer setup

Preparatory pressure response measurements were carried out in a circular Langmuir trough ($\pi$ 3.25 $cm^2 \times 0.5$ cm, ca. 16.6 mL) at constant area, as presented in Figs. 2 and 3 *a*. X-ray measurements with surface pressure control were carried out in a Langmuir trough (17 $\times$ 45 $\times$ 0.5 $cm^3$, ca. 400 mL) equipped with a movable Teflon barrier along the long axis of the trough (see Figs. 2 and 3 *b–e*). Additionally, to reach higher protein concentrations in the subphase, a small Langmuir trough (16 $\times$ 4 $\times$ 0.5 $cm^3$, ca. 32 mL) without a pressure-controlling barrier was used (see Figs. 5 and 6). Before every new filling, the troughs were thoroughly cleaned with isopropanol and ultrapure water. To check for remaining impurities, the trough was filled with ultrapure water, and the Teflon barrier was then closed to compress remaining impurities, which were subsequently sucked off with a vacuum pump. This process was repeated until no rise in surface pressure was observable during compression of a bare surface ($\Delta p < 0.2$ mN/m). The small trough was cleaned by carefully swiping a Teflon barrier over the entire surface, thus removing dirt from the surface of the subphase. The lipid compositions were chosen in view of the ability to form a stable monolayer with sufficient order for x-ray diffraction signals. At the same time the fraction of charged lipids should be comparable to that expected for the active zone. Note that an average of 12% charged lipids was reported in (19) for both leaflets. As PS lipids are typically asymmetrically distributed between the membrane leaflets (20), with the majority oriented toward the cytoplasm, we can expect concentrations of up to 24% at the inner leaflet, which we want to model here. For 1:1 PC/PS monolayers, equal mass concentrations of DPPC and DPPS were dissolved in a 20:9:1 mix of $CHCl_3$, MeOH, and water to yield a final lipid concentration of 0.2 mg/mL lipids in total. For the preparation of 19:1 PC/$PIP_2$ monolayers, 0.2 mg/mL DPPS and $PIP_2$ were dissolved in a 10:9:1 mix of the same solvents. Lipid stock solutions were carefully spread at different positions on the buffer surface with a microsyringe (Hamilton, NV, USA). About 200 μL and 60 μL were used for the large and small trough, respectively. The surface

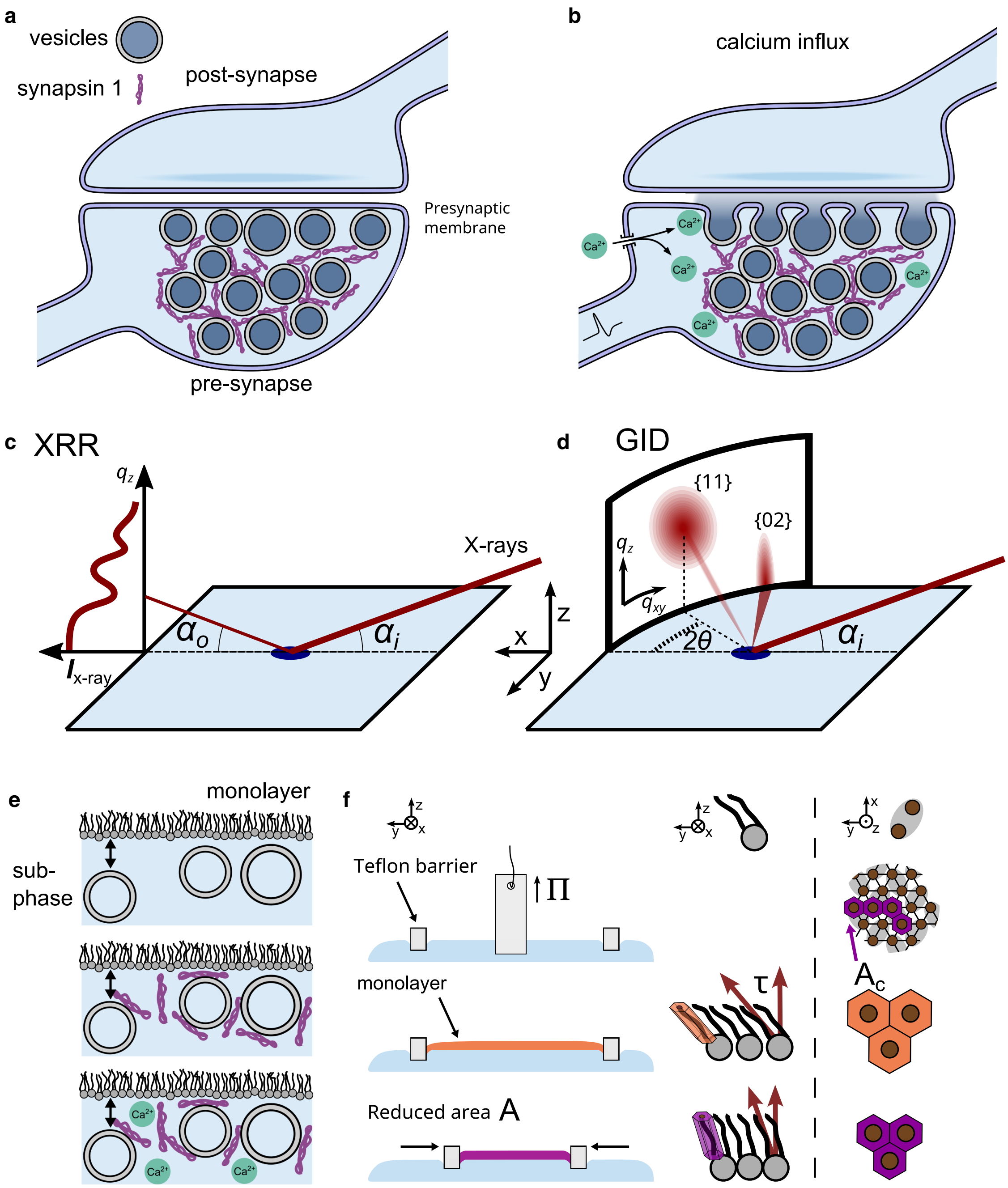


FIGURE 1 Experimental design and setup. (*a* and *b*) Schematic of the pre- and postsynapse, highlighting the role of synapsin in organizing the synaptic vesicle cluster (SVC). (*a*) The SVC acts as synaptic vesicle (SV) storage and supply unit in the presynapse. (*b*) Fusion and release of neurotransmitters into the synaptic cleft is triggered by a calcium influx. (*c*) X-ray reflectivity (XRR) configuration recording the specularly reflected beam from the interface $R(\alpha_i) = I_R(\alpha_i)/I_0$, as a function of angle of incidence $\alpha_i$. The measured curve is a transform of the interfacial density profile $\rho(z)$. (*d*) Grazing incidence diffraction (GID) configuration, with a fixed angle of incidence $\alpha_i = 0.79$ mrad. The out-of-plane reflections {11},{02} originating from the 2D crystalline ordering of acyl chains encode their tilt angle $\tau$ and the area per chain $A_c$. (*e*) In the synapse-free in vitro model the states in (*a*) and (*b*) are modeled by injecting vesicles, Syn1, and $Ca^{2+}$ into the subphase below a Langmuir monolayer. The presence/absence of Syn1 modulates the monolayers' interaction with vesicles and changes the film structure, i.e., the ordering of lipid chains and the respective parameters $A_c$, $\tau$, as well as the vertical density profile $\rho$. (*f*) Decreasing film area $A$ results in a change of acyl chain tilt angle, $\tau$, as well as the microscopic area per chain $A_c$.

pressure was measured with a calibrated Wilhelmy balance during all x-ray measurements in both troughs. Measurements were carried out at $\Pi$ = 10, 20, and 30 mN/m in the large trough. In the small trough, an initial monolayer surface pressure of approximately $\Pi$ = 20 mN/m was used.

## Synchrotron beamline

X-ray experiments on the Langmuir monolayer were carried out at beamline ID10 EH1 of the European Synchrotron Radiation Facility (ESRF) in Grenoble (21–23). The three ID10 undulators were tuned in series to

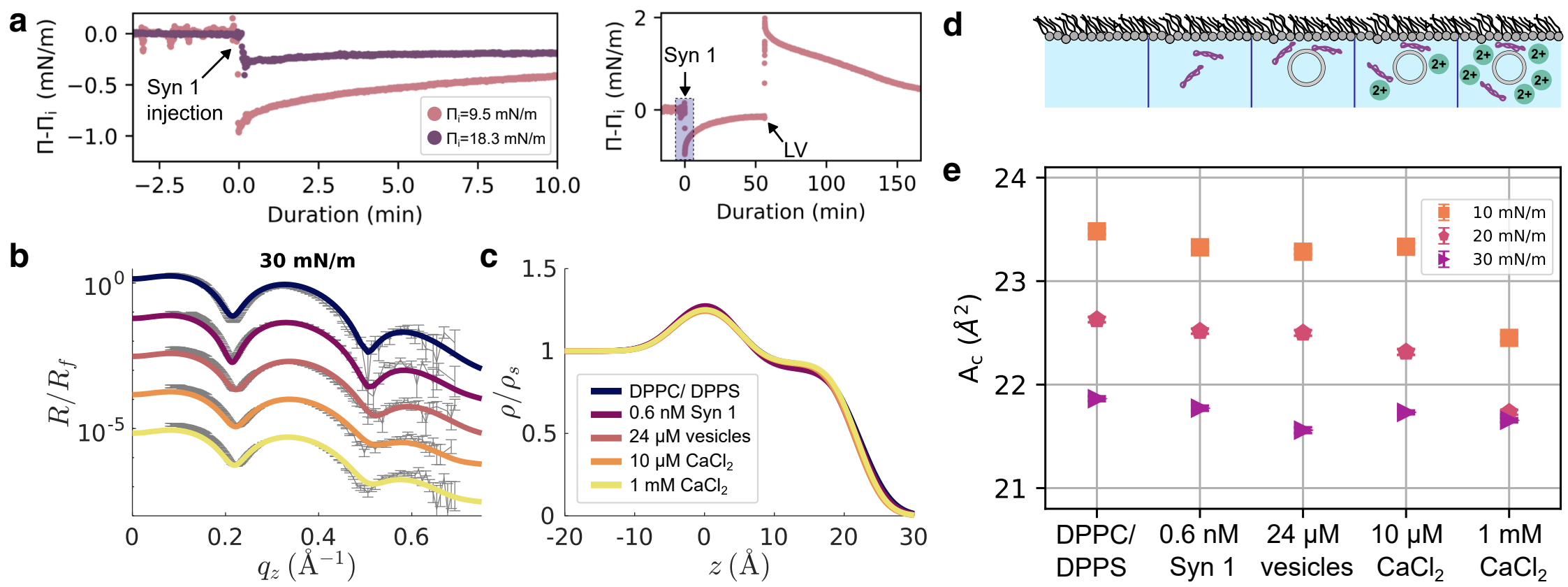


FIGURE 2 DPPC-DPPS (1:1) Langmuir monolayer pressure (*a*) and structure (*b–e*) response to the injection of Syn1, vesicles, and $CaCl_2$. Data in (*a*) were collected in a 16-mL trough with constant area and (*b–e*) in a 400-mL trough with constant pressure. (*a*) Monolayer pressure response to a 56.6-pM Syn1 injection at two different initial pressures, $\Pi_i = (9.5, 18.3)$ mN/m, and at constant area shows a negative response and subsequent partial relaxation (*left*). The relaxation remains partial, even after long timescales (*right*), and is specific to the synapsin injection, whereas vesicles produce a positive response (cf. Fig. 3). (*b*) XRR (*gray*) injection series taken at a constant pressure $\Pi = 30$ mN/m of 0.6 nM Syn1, 24 μM lipid vesicles, 10 μM $CaCl_2$, and 1 mM $CaCl_2$. Fits to the XRR reflectivity data (*gray*) at $\Pi = 30$ mN/m produced the density profiles in (*c*). They show no significant variation between the different systems. (*d*) shows a schematic illustration of the injection sequence and the monolayer. The corresponding GID results are given in (*e*) and similarly show only minor variations in the area per chain $A_c$. All concentrations are given relative to the subphase volume.

generate a synchrotron beam of high brilliance, which was subsequently monochromatized to 22 keV by an Si(111) channel-cut monochromator. The beam was focused by a set of 29 compound refractive lenses to a beam size of $25 \times 13$ μm$^2$ with a flux of approximately $5.7 \times 10^{12}$ ph/s in 16-bunch mode of the synchrotron source (70 mA nominal beam current). To enable GID and XRR measurements on liquid surfaces, the x-ray beam was tilted by a double-crystal deflector before impinging on the sample. The Langmuir trough was placed on an active antivibration stage and kept under a water-saturated helium atmosphere during measurements to reduce parasitic scattering effects. The trough table was connected to a water chiller with a set temperature of 18°C, leading to a trough temperature slightly below room temperature (approximately 20°C).

## Grazing incidence diffraction

For GID measurements, a linear position-sensitive Mythen 2K detector (Dectris, Baden, Switzerland) was mounted behind a Soller collimator with an in-plane angular resolution of 1.4 mrad. For each GID measurement, the angle of incidence was set to $\alpha_i = 0.79$ mrad, just below the critical angle $\alpha_c$. A single GID measurement comprised 126 measurements of

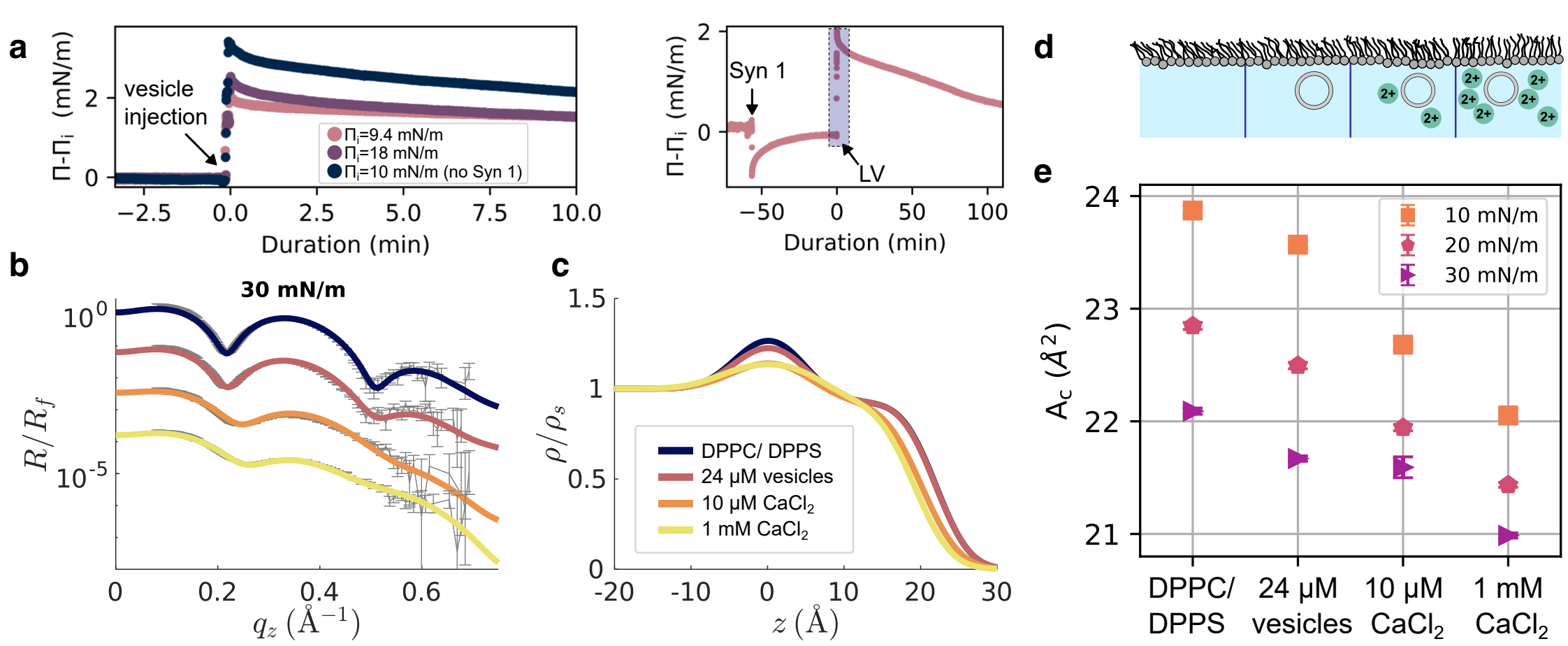


FIGURE 3 Response of the 1:1 DPPC-DPPS Langmuir monolayer interaction with lipid vesicles and $CaCl_2$ in absence of Syn1. (*a*) Pressure response $\Pi(t)$ relative to the initial pressure $\Pi_i$ upon an injection of lipid vesicles (23.5$\mu$ M lipids, 100 nm extrusion diameter) into the subphase. Rose and light-purple responses indicate the vesicle injection response to a subphase containing 56.6 pM Syn1. The response of an injection into a pure buffer subphase without Syn1 is shown in dark blue (*left*). The response leads to a continuous slow relaxation, even after long timescales (*right*) and is specific to the vesicle injection (*right*, same data as in Fig. 3 *a*). (*b*) XRR reflectivity data (*gray*) at $\Pi = 30$ mN/m fitted with a two-slab model following (3). The corresponding density profiles are shown in (*c*) and show a clear change in the monolayer upon injection of $CaCl_2$. A small effect leading to a slightly reduced headgroup density is also visible upon the vesicle injection. (*e*) GID data collected at $\Pi$=(30, 20, 10) mN/m of the initial monolayer and after subsequent injections at a constant pressure $\Pi = 30$ mN/m of vesicles ($d = 50$ nm, 24$\mu$ M lipids), 10$\mu$ M $CaCl_2$, and 1 mM $CaCl_2$, as illustrated in (*d*). Concentrations are given relative to subphase volume.

5 s at different values of the out-of-plane angle $2\theta$ or the lateral momentum transfer $q_{xy}$, correspondingly. Each separately recorded 1D intensity profile $I_{q_{xy}}(q_z)$ was then combined into a 2D intensity map $I(q_z,q_{xy})$, covering the in-plane and perpendicular components of the scattering vector $q_z = 4\pi/\lambda sin\alpha$ and $q_{xy} = 4\pi/\lambda sin\theta$, respectively (see Figs. S1–S3). The positions of the hydrocarbon lattice peaks were determined by least-square fitting, as detailed in the supporting material. From the peak positions, the area per chain $A_c$ can be calculated from the unit cell parameters $a = 1/\sqrt{d_{11}^{-2} - (2d_{02})^{-2}}$ and $b = 2d_{02}$ (24), which are obtained from the in-plane components $q_{xy}^{hk} = 2\pi/d_{hk}$ of the corresponding lattice peaks (see supporting material). The area per chain is then given by (25)

$$A_c = \frac{ab}{2} = \frac{d_{02}}{\sqrt{d_{11}^{-2} - (2d_{02})^{-2}}}. \quad (1)$$

Here, the nonzero vertical components $q_z^{11}$ of the $d_{11}$ peaks indicate that the lipid chains have a nearest neighbor tilt with respect to the surface normal of the Langmuir monolayer (26). The tilt angle $\tau$ follows from $q_z^{11}$ and the in-plane components $q_{xy}^{11}$ and $q_{xy}^{02}$ (26,27)

$$\tan(\tau) = \frac{q_z^{11}}{\sqrt{\left(q_{xy}^{11}\right)^2 - \left(\frac{q_{xy}^{02}}{2}\right)^2}}. \quad (2)$$

The tilt angles $\tau$ are shown in the supporting material. Since they are highly correlated with $A_c$, we have included only the latter in the result graphics of the main manuscript.

## X-ray reflectivity

For XRR measurements, a 2D CdTe Maxipix 2x2 photon counting pixel detector was used. To reduce stray scattering, an evacuated flight tube with two sets of collimating slits at the entry and exit points of the tube was used to select in-plane specularly reflected photons. Raw data processing to adjust for attenuators and background was performed using in-house developed scripts at the beamline. Fits to the recorded XRR were performed with a combination of a deterministic simplex algorithm (15) and a stochastic sampling of initial fit parameters to test for the stability of the fit result (see supporting material for more details). To facilitate fitting, the reflectivity data was divided by the Fresnel reflectivity $R_F = |(q_z - q'_z)/(q_z + q'_z)|^2$, where $q_z'^2 = q_z^2 - q_c^2$ where $q_c$ denotes the critical angle to correct for refraction at the interface. The density profiles of the water-lipid-air interface $\rho(z)$ were modeled using a two-box model with densities $\rho_{h,t}$ and width $L_{h,t}$ for the lipid head and tail. The density transition profile was then built from a sum of error functions with the additional interface roughness parameter $\sigma$. To avoid overfitting, we chose to set $\sigma_i = \sigma$ equal for all three interfaces (subphase-head, head-tail, tail-air) while keeping individual parameters for density and length of head and tail groups. The corresponding reflectivity curve was then calculated from the density profile $\rho(z)$ according to (28)

$$R(q_z) = R_F(q_z)\left|\int \frac{d\rho(z)}{dz} e^{-iq_{zz}} dz\right|^2. \quad (3)$$

Generally, we found the fitting to lack robustness in the sense that almost similar $\chi^2$ values can be obtained for different model parameters. We therefore imposed further constraints on the profiles, requiring the chain density to be below 93% of the subphase density, as detailed in the supporting material.

**TABLE 1 Synapsin 1 concentrations for the presented data**

| Figure | Syn1 | Increase |
|---|---|---|
| Figs. 2 and 3 *a* | 0.0566 nM | |
| Fig. 2 *b–e* | 0.6 nM | 10.6 |
| Figs. 5 and 6 | 8 nM | 13.3 |

## RESULTS

### Interaction of synapsin with lipid monolayers composed of DPPC/DPPS

Fig. 2 shows the response of the monolayer upon subsequent addition of synapsin 1 (Syn1, see Table 1), LVs, and two different concentrations of $CaCl_2$, as illustrated in Fig. 2 *d*. Between each injection, the pressure was reset to 30 mN/m, and the trough was left to equilibrate. Changes in the film are monitored by recording the surface pressure $\Pi$ (Fig. 2 *a*) and by determining the vertical electron density profile $\rho(z)$ (Fig. 2 *b* and *c*) and the area per lipid chain $A_c$ (Fig. 2 *e*) from XRR and GID measurements, respectively. All measurements were taken at three subsequent surface pressures (30, 20, 10 mN/m). The data has been recorded using (Fig. 2 *a*) the small and (Fig. 2 *b–e*) the large Langmuir trough (see materials and methods section). After injection of Syn1, the surface pressure traces $\Pi(t)$ plotted in Fig. 2 *a* exhibit a step-like decrease followed by partial relaxation. The decrease is most pronounced for initial pressure $\Pi_i$ = 10 mN/m and becomes weaker for higher $\Pi_i$. Note that the traces of $\Pi$ differ from control experiments where pure buffer was injected instead of Syn1 (not shown). In this case, a transient perturbation in the film is typically observed in the form of a sharp spike that relaxes almost instantaneously. The XRR data with least-square fit (solid curve), and the corresponding density profiles $\rho(z)$, are shown in Fig. 2 *b* and *c*, for $\Pi_i$ = 30 mN/m. Note that meaningful XRR results require the monolayer to be sufficiently compressed, presumingly since phase coexistence and incoherent averaging otherwise reduces the reflectivity modulations. We therefore have evaluated only the XRR data at highest compression ($\Pi_i$ = 30 mN/m). The fit parameters including the length of the headgroup $l_h$, the lipid tails $l_t$, and the corresponding densities $\rho_h$ and $\rho_t$ are tabulated in Table 2, together with the mean-square error of the fit $\chi^2$. The parameters and the associated density profiles do not show any significant changes as Syn1 is injected into the subphase.

**TABLE 2 Parameter values of the XRR fits for $\Pi$ = 30 mN/m (data and fits shown in Fig. 2 *b* and *c*)**

| | $\sigma$ | $\rho_h/\rho_s$ | $l_h$(Å) | $\rho_t/\rho_s$ | $l_t$(Å) | $I_0$ | $\chi^2$ |
|---|---|---|---|---|---|---|---|
| DPPC/DPPS | 3.56 | 1.35 | 8.56 | 0.92 | 17.59 | 1.38 | 163.42 |
| 0.6 nM Syn1 | 3.41 | 1.35 | 9.06 | 0.89 | 17.22 | 1.21 | 32.46 |
| 24 $\mu$M vesicles | 3.30 | 1.31 | 8.92 | 0.93 | 16.77 | 1.20 | 291.47 |
| 10 $\mu$M $CaCl_2$ | 3.18 | 1.28 | 9.66 | 0.92 | 16.48 | 1.17 | 229.81 |
| 1 mM $CaCl_2$ | 3.18 | 1.29 | 9.59 | 0.93 | 16.77 | 1.11 | 258.78 |

Next, we turn to the area per headgroup $A_c$ shown in Fig. 2 *e* for all three pressures $\Pi_i$. As expected for Langmuir isotherms, $A_c$ decreases with increasing $\Pi_i$ (26).

Contrarily, the injection of 0.6 nM Syn1 and of 24 μM LVs results in no significant change or only a minor decrease in the case of the highest surface pressure $\Pi = 30$ mN/m. Similarly, the subsequent injection of 10 μM of $CaCl_2$ into the subphase, mimicking synaptic calcium influx, also results in very small changes, namely a slight decrease of $A_c$ for 20 mN/m film and an increase for 30 mN/m. Major changes are only observed upon the injection of the unphysiologically large amount of 1 mM $CaCl_2$ into the subphase at 10 and 20 mN/m. The negligible effect in the first measurement shown in Fig. 2 *e* might be due to incomplete equilibration at the time of the measurement. These reductions of $A_c$ indicate a compaction of the monolayer presumingly due to the strong electrostatic interaction of $Ca^{2+}$ and the anionic monolayer. This is in line with the electron density profile at this high $CaCl_2$ concentration, which also indicates a slightly more compact structure.

The corresponding control experiment without a Syn1 injection is shown in Fig. 3. In contrast to the measurements in the presence of Syn1 in the subphase, both GID as well as XRR measurements show large changes after each injection. As vesicles are added into the subphase, the area per chain decreases by approximately 0.4 $Å^2$ ($\approx 2\%$) across all surface pressures Π, suggesting either fusion or adhesion of the vesicles to the monolayer. A further decrease is observed after the injection of 10 μM $CaCl_2$ and similarly after injection of 1 mM $CaCl_2$. In view of the 100-fold concentration increase, a decrease of similar amplitude may seem surprising. We can attribute this to saturation, already at moderate subphase concentrations. It seems likely that this saturation is brought about by the limited availability of LVs (24 μM in the bulk). If this reservoir is depleted, added $CaCl_2$ cannot recruit more vesicles to the interface. The results of the XRR measurements (Fig. 3 *c* and *d*), again evaluated at 30 mN/m, exhibit a washing-out of the second minimum of the reflectivity curve and, correspondingly, a decrease in headgroup density after vesicle injection (see also parameters tabulated in Table 3). When $CaCl_2$ is injected into the subphase, the headgroup density further decreases, and the monolayer thickness changes. Compared with the XRR measurements with Syn1, the effects observed here are much more pronounced. Note that the GID data indicate a reduction in tilt angle, which should go along with an increased film thickness. This apparent paradox can be resolved when one keeps in mind that the XRR signal averages over the entire film including liquid expanded and liquid ordered domains, which GID signals relate only to the latter. Furthermore, shorter lipids such as cholesterol may be incorporated in the lipid expanded domains after vesicle injection.

**TABLE 3 Parameter values of the XRR fits for $\Pi = 30$ mN/m (data and fits shown in Fig. 3 *b* and *c*)**

| | $\sigma$ | $\rho_h/\rho_s$ | $l_h$(Å) | $\rho_t/\rho_s$ | $l_t$(Å) | $I_0$ | $\chi^2$ |
|---|---|---|---|---|---|---|---|
| DPPC/DPPS | 3.50 | 1.35 | 8.75 | 0.93 | 17.25 | 1.13 | 46.13 |
| 24 $\mu$M vesicles | 3.55 | 1.28 | 9.38 | 0.93 | 16.86 | 1.28 | 52.32 |
| 10 $\mu$M $CaCl_2$ | 3.91 | 1.18 | 10.11 | 0.92 | 14.43 | 1.37 | 23.93 |
| 1 mM $CaCl_2$ | 3.80 | 1.15 | 13.41 | 0.92 | 11.93 | 1.28 | 24.26 |

The comparison, hence, clearly indicates that the presence of Syn1 (prior incubation of the film) greatly reduces the interaction of vesicles with the DPPC/DPPS monolayer. This difference is most striking when comparing the GID results, i.e., Figs. 3 *e* and 2 *e*, respectively. Syn1 seems to suppress major structural changes of the film by modulating the interactions with vesicles and $Ca^{2+}$. Only when the concentration of divalent $Ca^{2+}$ cations is extremely high (1 mM), a noticeable structural change is observed. The presence of Syn1 limits also the direct interaction between vesicles and the monolayer, without $Ca^{2+}$ addition, and also independently of surface pressure $\Pi_i$. Altogether, both GID and XRR results support the interpretation that Syn1 acts as a buffering agent, screening possible interactions from the monolayer.

This "structural buffering" may result from the N-terminus of Syn1 binding to the anionic monolayer, driven by electrostatic interactions. Steric forces of the intrinsically disordered region of Syn1 could at the same time prevent LVs from coming into close proximity of the monolayer, or at least from interacting too strongly with the monolayer, for example by tight docking or fusion. The previously reported clustering of LVs (6,12) does not seem to be a suitable explanation for the observed screening effect, as this effect should only become visible upon injection of LVs into the subphase. The significant interaction between the protein itself and the monolayer suggests a direct screening, for example by forming a protective protein layer. We cannot exclude, however, that Syn1-induced clustering of vesicles could also contribute to the effect by confining the vesicles to condensates below the film and thereby preventing uniform interactions with the monolayer. In this scenario, only a few vesicles would be available for potential interaction at the interface between the monolayer and a vesicle cluster. This could also explain the fact that in the presence of Syn1, the response of $\Pi(t)$ to vesicle injection does not seem to depend on the monolayer compression states. In fact, at lower compression, when liquid-ordered and liquid-disordered phases coexist, one would expect stronger changes, as lipids originating from vesicles can insert themselves more easily into a liquid-disordered phase, leading to a rearrangement of the entire monolayer. When Syn1 is present, and able to recruit vesicles into condensates in the subphase or beneath the liquid ordered phase, vesicle interaction with the less ordered phase could possibly be depleted.

## Extending the model system: $PIP_2$ and synaptic vesicles

Next, we investigate possible specific effects caused by interaction of synapsin with $PIP_2$ and also with SVs at the

interface. Due to the limited availability of the protein, a higher concentration of synapsin in the subphase could only be achieved using a smaller trough (sample volume 32 mL). Due to its small size, this trough was not equipped with a movable barrier, such that all measurements were carried out at a constant area rather than at constant pressure. Injections of Syn1 and vesicles into the subphase could therefore not be performed while adjusting the barrier to keep Π constant. Thus, different trajectories in $A_c$/Π space are possible after injection, as indicated schematically in Fig. 4. In the simplest case, the monolayer is condensed and $A_c$ decreases with increasing surface pressure, for example when more lipid molecules are added to the monolayer. For the small variations in pressure considered here, this isothermic relation can be linearized. Contrarily, jumps away from the isotherm, for example isobaric and isochoric transitions, are indicative for changes in monolayer composition and interactions, e.g., by insertion of a transmembrane domain or a different type of lipids. In the present case, for example, local interactions of Syn1 and vesicles in close proximity to the monolayer, as well as a reorganization of different domains in the film, i.e., patches of different lipid packing, could cause changes in the equation of state.

The effect of an increased amount of Syn1 in the subphase on the same sequence of injections as in the previous section (24 μM LV, 10 μM $CaCl_2$) is shown in Fig. 5 *a*. The 1:1 DPPC/DPPS monolayer responds with a nearly isobaric contraction to the synapsin injection, an isothermic compression to the vesicle injection, and a further isobaric compression after the $CaCl_2$ injection. The isobaric decrease of $A_c$ upon the Syn1 injection aligns well with the findings from Fig. 2 *e* at 20 mN/m. Comparing the effect for small and large troughs, we infer that an approximately 20-fold increase in Syn1 concentration only leads to an approximately four times stronger reduction of $A_c$ (cf. Figs. S2 and S3). The reduction of $A_c$ upon injection of 10 μM $CaCl_2$ is larger than when the same amount was injected at lower synapsin concentration. Assuming that $CaCl_2$ promotes membrane-vesicle interaction, a higher Syn1 concentration could possibly reduce the number of LVs, which are free to interact with the monolayer, for example by sequestering vesicles in subphase clusters.

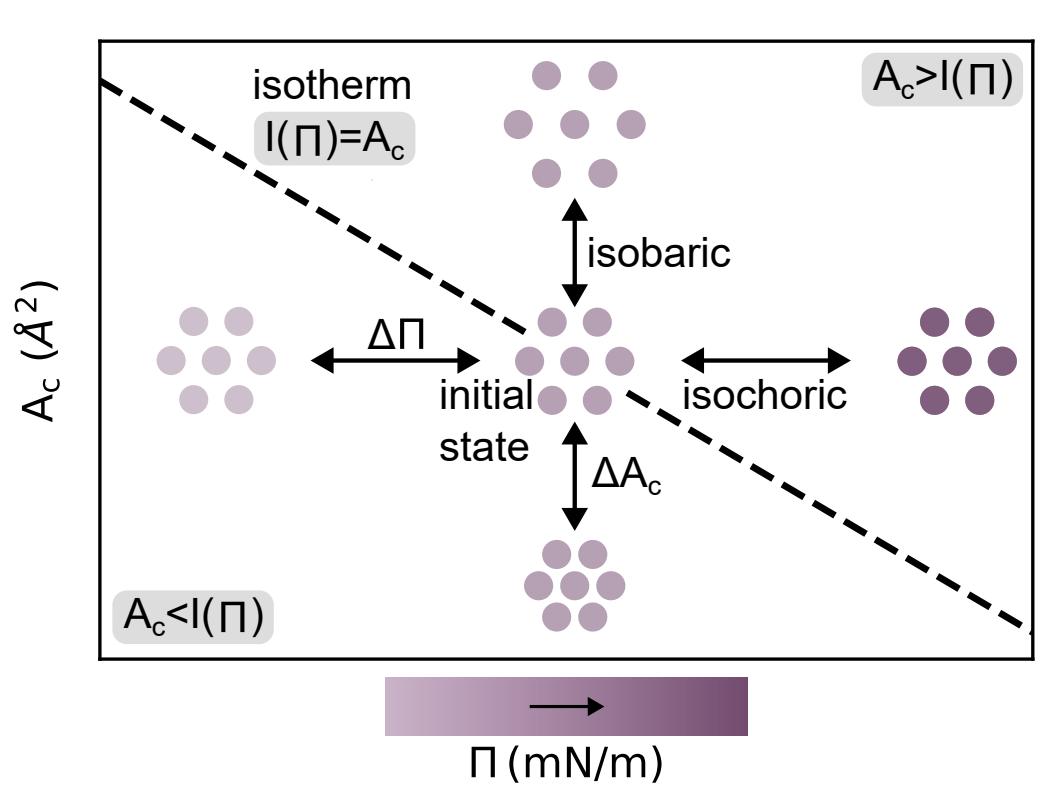


FIGURE 4 Conceptual guide for isothermal, isobaric, and isochoric transitions in the $A_c$-Π plane, with a dashed reference isotherm.

In a second set of measurements, shown in Fig. 5 *b*, the same sequence was repeated with a monolayer composed of 19 DPPC:1 $PIP_2$. The approximate $PIP_2$ headgroup charge of −4 at pH 7.4 leads to a more spaced-out, but locally more strongly charged, monolayer with an overall charge density that is comparable to the 1:1 DPPC/DPPS monolayer where each phosphatidylserine (PS) headgroup carries a single negative charge (average charge −0.2, compared with −0.5 in DPPC/DPPS) (29). The DPPC/$PIP_2$ monolayer composition can be considered as a model of the plasma membrane near the active zone, as $PIP_2$ is found in abundant quantities at the active zone and as a key player in exocytosis in the synapse (30). In contrast to the DPPC/DPPS monolayer, only minor deviations from the reference isotherm arise in the DPPC/$PIP_2$ monolayer upon injection of Syn1, LV, and $CaCl_2$, as shown in Fig. 5 *b*. Variations in $A_c$ across all four measurements span approximately 0.2 Å$^2$, whereas the same set of measurements in Fig. 5 *a* span more than 1 Å$^2$. The slow relaxation of Π(*t*) to the injection of Syn1 and vesicles shows that the observed isothermic change is likely due to diffusion-limited interactions of these components with the monolayer. In contrast to the DPPC/DPPS monolayer, these interactions are, however, isothermic and likely caused by a compaction of the monolayer due to the presence of additional lipids or proteins at the interface. These changes, hence, do not seem to be induced by a reorganization of the domains in the film or monolayer structure, as possibly the case for isobaric changes in the DPPC/DPPS monolayer. The lower overall charge density of 5% $PIP_2$ in the monolayer appears to be insufficient for a pronounced interaction with Syn1, despite higher local charge state. Although the increased concentration of $PIP_2$ at the active zone may have strong effects on the interaction with synaptic vesicles, it does not seem to strongly modulate the interaction with Syn1 at the interface.

In the two final sets of measurements carried out with the small trough in view of limiting sample consumption, we substituted the LVs with purified SVs, which still carry the full set of SV-associated proteins (18). The results are presented in Fig. 6 *a* for a DPPC/DPPS monolayer and in Fig. 6 *b* for a DPPC/$PIP_2$ monolayer. The DPPC/$PIP_2$-based system shows a significant isobaric decrease of $A_c$ as SVs are injected into the subphase, followed by an isothermic state change when Syn1 is injected. A similar response to the injection of SVs in the DPPC/$PIP_2$ system is also observed in the DPPC/DPPS system, followed by a pronounced isothermic reduction of surface pressure by approximately 4 mN/m upon addition of Syn1 into the subphase. At the time of the GID measurement, the Π(*t*) has already partially relaxed. The final $CaCl_2$ injection again results in approximately the same shift toward higher Π and lower $A_c$, as it did in the same system with LV. Hence, we can

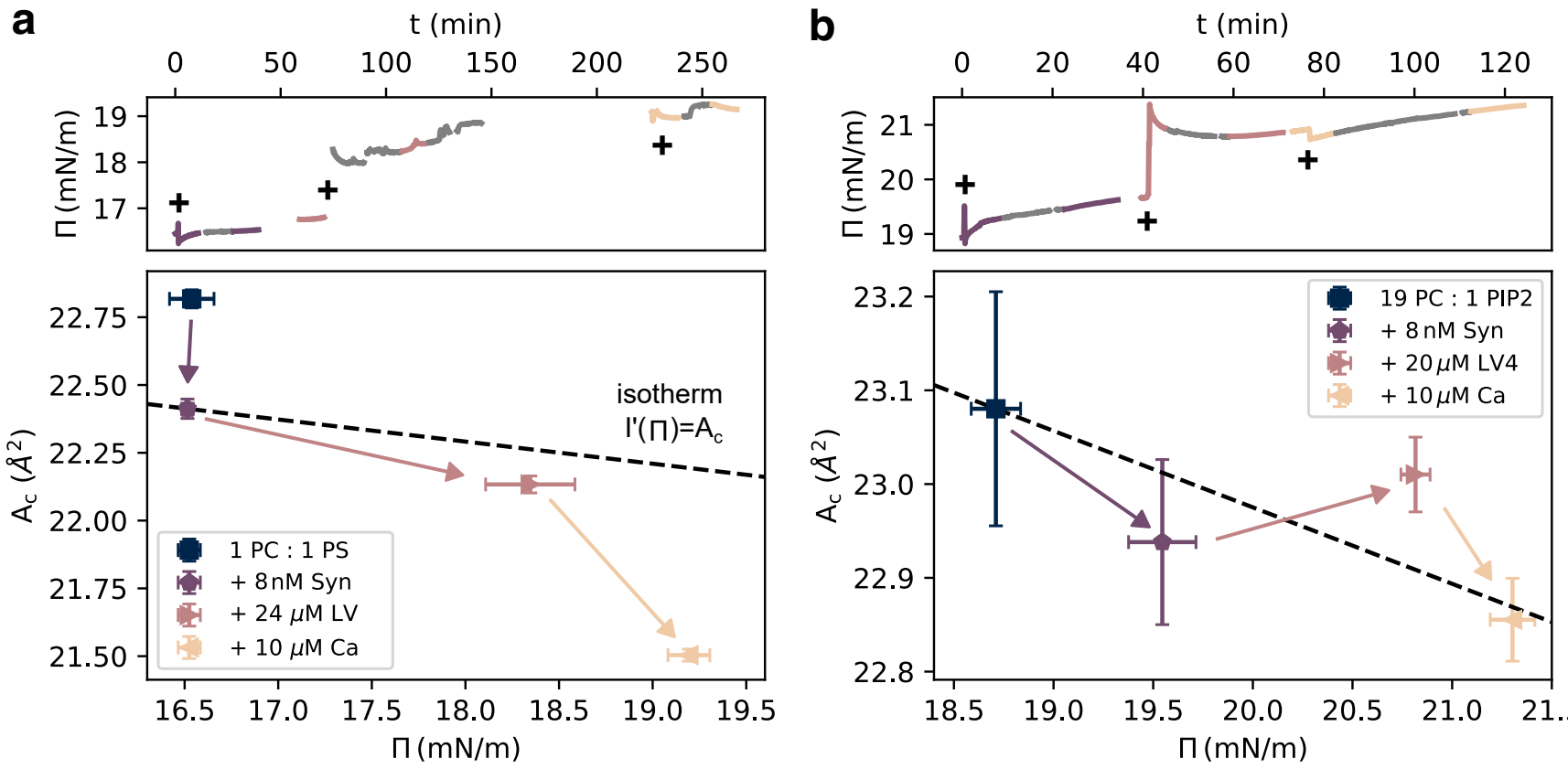


FIGURE 5 Injection of Syn1 at larger subphase concentrations (32 mL trough volume) increases the previously weak $A_c$ reduction. Monolayer structural changes are plotted in the $A_c$-$\Pi$ plane for subsequent injection steps. The upper parts indicate the change of pressure during the time of the experiment. Injections are marked with a + sign. (*a*) Injection of approximately 8 nM Syn1 into an otherwise equal injection sequence as compared with Fig. 2. The isobaric decrease of $A_c$ after Syn1 and $CaCl_2$ injections is now clearly visible. (*b*) The effect vanishes when the 1 DPPC:1 DPPS monolayer is exchanged for a monolayer of 19 DPPC:1 $PIP_2$ that carries a lower average charge but a higher charge per molecule of $PIP_2$.

conclude that for DPPC/PS, the injection of LV and SV results in similar changes, likely to be caused by strong electrostatic interaction, whereas the two vesicle systems differ when interacting with DPPC/$PIP_2$. This difference is plausible in view of the specific interaction of SV proteins with $PIP_2$, notably synaptotagmin (31).

Note that injection of Syn1 into the subphase of the DPPC/$PIP_2$ monolayer results in an increase of surface pressure, rather than the decrease observed for DPPC/PS. We do not consider the pressure increase to be a direct effect of synapsin, but of the recruitment of SVs to the interface by synapsin, resulting in transfer of lipids. This effect seems to depend on the presence of $PIP_2$ and SVs.

## DISCUSSION

In summary, the system displays rather multifaceted and complex interactions, where the structural response of the monolayer to vesicle injection depends on the monolayer composition, the presence of Syn1, and the type of vesicle (lipid/synaptic). Although we cannot explain all observations in detail, notably the directions and amplitudes of the jumps observed in $A_c$-$\Pi$ space that are likely to be very sensitive to the equilibration after an injection, we can draw a few major conclusions.

- Injection of Syn1 into the subphase results in a decrease of surface pressure $\Pi$ (Fig. 2 *a*).
- Injection of vesicles into the subphase results in an increase of surface pressure $\Pi$, most likely by partial transfer of lipids from vesicles into the monolayer, until the chemical potential equilibrates (Fig. 3 *a*).
- The presence of Syn1 at the monolayer interface buffers further interaction of subphase components with the monolayer and makes it less susceptible to changes upon further injection of vesicles and $Ca^{2+}$ (Fig. 2).
- The high and rather uniform charge density of the monolayer is crucial for the interaction with Syn1 (Fig. 5).
- XRR measurements are not sensitive enough to predict a separate layer of protein or vesicles near the interface, despite the significant influence of both components on the monolayer, as shown by both GID and XRR measurements (Figs. 2 *c* and 3 *c*).
- Changes in monolayer thickness and headgroup density are, however, shown to be influenced by the injection of vesicles and $Ca^{2+}$ ions into the monolayer, an effect that is absent in the presence of Syn1 in the monolayer.

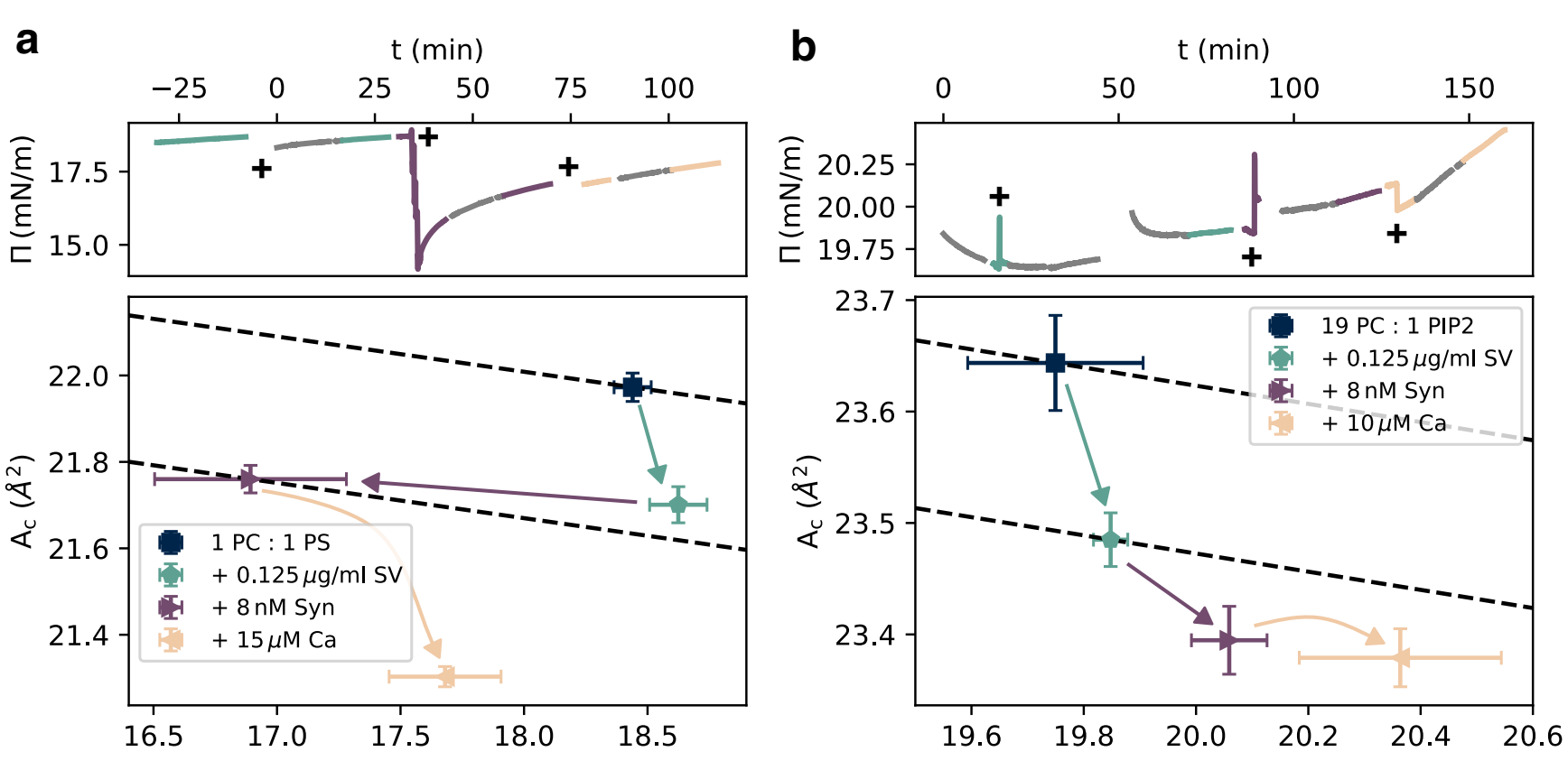


FIGURE 6 Effect of the injection of synaptic vesicles (SVs) on the interaction of synapsin with a monolayer. Measurements were taken on a 32-mL trough, and results are shown in the $\Pi$-$A_c$ plane. Traces at the top indicate the change of pressure during the experiment. Injections are marked with a + sign. (*a*) Injection of approximately 0.125 μg/mL of SVs into a 1 DPPC:1 DDPS monolayer system shows a broadly similar behavior as the corresponding lipid vesicle injection also upon further injection of synapsin and $CaCl_2$. The isothermic shift toward higher $\Pi$ is an effect of incomplete equilibration but qualitatively similar to Fig. 5 *a*. (*b*) In a 19 DPPC:1 $PIP_2$ system, SV injection leads to an isobaric change in $A_c$ that was not observed after a lipid vesicle injection.

Certainly, this study could be followed by several further improvements and extensions. Firstly, the set of experiments, regarding in particular composition, concentration, and sequence of injections, could be extended and completed. For example, the monolayer composition could be extended to model more accurately the active zone composition given in (19). On the technical side, regarding XRR, a larger range of $q_z$ could help to ensure that the least-square fitting gives more robust and unique density profiles. At the same time, a model involving lateral domains (modeled by incoherent addition of reflectivity signals) would overcome the restrictive approximation of a uniform structure. Note, that for GID, this approximation is not so restrictive, as contributions from loosely ordered and less dense domains can be neglected. Furthermore, a combination of XRR and neutron reflectivity could be very beneficial and would allow placing further constraints on the fit model. Extensions of this study could further include a second synaptic protein, $\alpha$-synuclein, which was already demonstrated to couple to Langmuir monolayers (32) and flat lipid bilayers (33). Finally, the formation of eventual lateral domains could be investigated more closely by Brewster microscopy or fluorescence microscopy (for labeled synapsin) in combination with GUVs. Notwithstanding further extensions, the present study has already revealed the protective role of synapsin 1 for the monolayer structure. In the biological context of the synapse, this "protective role" may prevent unwanted perturbations or fusion at the synaptic membrane, as long as specific interactions involving $Ca^{2+}$ and vesicle proteins are not triggered.

## DATA AND CODE AVAILABILITY

The data of the synchrotron experiments presented here collected at ESRF beamtime SC-5361 have been deposited under Database: https://doi.esrf.fr/10.15151/ESRF-ES-886072026 at the ESRF data portal and are publicly available. Analysis scripts are available under Databank (GRO: data): https://doi.org/10.25625/4QDVWC.

## ACKNOWLEDGMENTS

This manuscript is dedicated to late Prof. Erich Sackmann, who had pioneered supported lipid membranes and monolayers and who kindly offered mentorship to the senior author of this work when starting projects in membrane biophysics. The authors would like to thank M. Ganzella and R. Jahn for providing us with purified synaptic vesicles. We thank the ESRF for allocation of beamtime SC-5361 at ID10 (09/22). H.B., T.C., and T.S. thank the Partnership for Soft Condensed Matter (PSCM) for hosting them during all preliminary experiments and for providing all necessary chemicals as well as support laboratories before and during the beamtime. We thank Oleg Konovalov for his expert advice during the preparation of the beamtime and Pierre Lloria for his help with the setup of preliminary Langmuir trough experiments. Beamtime data is available at Database: https://doi.org/10.15151/ESRF-ES-886072026. The project has been funded through grants SFB1286/A2 and Project-ID 449750155 – RTG 2756, Project B2 of the German science foundation (DFG).

## AUTHOR CONTRIBUTIONS

T.S., D.M., H.B., and T.C. designed research. H.B., T.C., C.N., M.J., D.P., and T.S. performed synchrotron experiments. C.H. and D.M. purified and contributed proteins, and H.B. and T.C. performed data analysis. H.B., T.C., and T.S. wrote the article.

## DECLARATION OF INTERESTS

The authors declare no competing interests.

## SUPPORTING MATERIAL

Supporting material can be found online at https://doi.org/10.1016/j.bpj.2025.12.004.

## REFERENCES

1. Cesca, F., P. Baldelli, …, F. Benfenati. 2010. The synapsins: Key actors of synapse function and plasticity. *Prog. Neurobiol.* 91:313–348. https://doi.org/10.1016/j.pneurobio.2010.04.006.
2. Wilhelm, B. G., S. Mandad, …, S. O. Rizzoli. 2014. Composition of isolated synaptic boutons reveals the amounts of vesicle trafficking proteins. *Science.* 344:1023–1028.
3. Katz, B. 1971. Quantal mechanism of neural transmitter release. *Science.* 173:123–126.
4. Sansevrino, R., C. Hoffmann, and D. Milovanovic. 2023. Condensate biology of synaptic vesicle clusters. *Trends Neurosci.* 46:293–306. https://doi.org/10.1016/j.tins.2023.01.001.
5. Reshetniak, S., C. A. Bogaciu, …, S. O. Rizzoli. 2024. The synaptic vesicle cluster as a controller of pre-and postsynaptic structure and function. *J. Physiol.* 603:5935–5964.
6. Milovanovic, D., Y. Wu, …, P. De Camilli. 2018. A liquid phase of synapsin and lipid vesicles. *Science.* 361:604–607.
7. Hoffmann, C., J. Rentsch, …, D. Milovanovic. 2023. Synapsin condensation controls synaptic vesicle sequestering and dynamics. *Nat. Commun.* 14:6730.
8. Pechstein, A., N. Tomilin, …, O. Shupliakov. 2020. Vesicle clustering in a living synapse depends on a synapsin region that mediates phase separation. *Cell Rep.* 30:2594–2602.e3.
9. Cheetham, J. J., J. Murray, …, L. J. Johnston. 2003. Interaction of synapsin I with membranes. *Biochem. Biophys. Res. Commun.* 309:823–829.
10. Krabben, L., A. Fassio, …, V. Haucke. 2011. Synapsin I Senses Membrane Curvature by an Amphipathic Lipid Packing Sensor Motif. *J. Neurosci.* 31:18149–18154.
11. Ho, M. F., M. Bähler, …, P. Greengard. 1991. Synapsin I is a highly surface-active molecule. *J. Biol. Chem.* 266:5600–5607.
12. Alfken, J., C. Neuhaus, …, T. Salditt. 2024. Vesicle condensation induced by synapsin: condensate size, geometry, and vesicle shape deformations. *Eur. Phys. J. E Soft Matter.* 47:8.
13. Neuhaus, C., J. Alfken, …, T. Salditt. 2024. Morphology and intervesicle distances in condensates of synaptic vesicles and synapsin. *Biophys. J.* 123:4123–4134.
14. Stefaniu, C., and G. Brezesinski. 2014. X-ray investigation of monolayers formed at the soft air/water interface. *Curr. Opin. Colloid Interface Sci.* 19:216–227.
15. Ghosh, S. K., S. Castorph, …, T. Salditt. 2010. In vitro study of interaction of synaptic vesicles with lipid membranes. *New J. Phys.* 12:105004. https://doi.org/10.1088/1367-2630/12/10/105004.
16. Ghosh, S. K., S. Castorph, …, M. Holt. 2012. Measuring Ca2+-induced structural changes in lipid monolayers: implications for synaptic vesicle exocytosis. *Biophys. J.* 102:1394–1402.

17. Hoffmann, C., R. Sansevrino, …, D. Milovanovic. 2021. Synapsin Condensates Recruit alpha-Synuclein. *J. Mol. Biol.* 433:166961.

18. Takamori, S., M. Holt, …, R. Jahn. 2006. Molecular Anatomy of a Trafficking Organelle. *Cell.* 127:831–846. https://www.sciencedirect.com/science/article/pii/S0092867406014000.

19. Borgmeyer, M., C. Coman, …, R. Ahrends. 2021. Multiomics of synaptic junctions reveals altered lipid metabolism and signaling following environmental enrichment. *Cell Rep.* 37:109797.

20. Leventis, P. A., and S. Grinstein. 2010. The distribution and function of phosphatidylserine in cellular membranes. *Annu. Rev. Biophys.* 39:407–427.

21. Smilgies, D.-M., N. Boudet, …, O. Konovalov. 2005. Troika II: a versatile beamline for the study of liquid and solid interfaces. *J. Synchrotron Radiat.* 12:329–339. https://doi.org/10.1107/S0909049505000361.

22. Jankowski, M., V. Belova, …, A. Pastore. 2023. The complex systems and biomedical sciences group at the ESRF: Current status and new opportunities after extremely brilliant source upgrade. *Nucl. Instrum. Methods Phys. Res. B.* 538:164–172. https://www.sciencedirect.com/science/article/pii/S0168583X23000708.

23. Konovalov, O., V. Rein, …, M. Jankowski. 2024. Tripling of the scattering vector range of X-ray reflectivity on liquid surfaces using a double-crystal deflector. *J. Appl. Crystallogr.* 57:258–265. https://doi.org/10.1107/S1600576724000657.

24. Marsh, D. 2012. Lateral order in gel, subgel and crystalline phases of lipid membranes: Wide-angle X-ray scattering. *Chem. Phys. Lipids.* 165:59–76.

25. Ropers, M.-H., and G. Brezesinski. 2013. Lipid ordering in planar 2D and 3D model membranes. *Soft Matter.* 9:9440–9448.

26. Kaganer, V. M., H. Möhwald, and P. Dutta. 1999. Structure and phase transitions in Langmuir monolayers. *Rev. Mod. Phys.* 71:779–819. https://doi.org/10.1103/RevModPhys.71.779.

27. Andreeva, T. D., J. G. Petrov, …, H. Moehwald. 2008. Structure of the Langmuir monolayers with fluorinated ethyl amide and ethyl ester polar heads creating dipole potentials of opposite sign. *Langmuir.* 24:8001–8007.

28. Als-Nielsen, J., and D. McMorrow. 2011. Elements of Modern X-Ray Physics. John Wiley & Sons.

29. McLaughlin, S., J. Wang, …, D. Murray. 2002. PIP2 and proteins: interactions, organization, and information flow. *Annu. Rev. Biophys. Biomol. Struct.* 31:151–175.

30. Koch, M., and M. Holt. 2012. Coupling exo- and endocytosis: an essential role for $PIP_2$ at the synapse. *Biochim. Biophys. Acta.* 1821:1114–1132.

31. Park, Y., J. B. Seo, …, R. Jahn. 2015. Synaptotagmin-1 binds to PIP2-containing membrane but not to SNAREs at physiological ionic strength. *Nat. Struct. Mol. Biol.* 22:815–823.

32. Chaari, A., H. Horchani, …, M. Ladjimi. 2013. Surface behavior of $\alpha$-Synuclein and its interaction with phospholipids using the Langmuir monolayer technique: A comparison between monomeric and fibrillar $\alpha$-Synuclein. *Int. J. Biol. Macromol.* 58:190–198.

33. Dubackic, M., Y. Liu, …, U. Olsson. 2022. $\alpha$-Synuclein Interaction with Lipid Bilayer Discs. *Langmuir.* 38:10216–10224.

Biophysical Journal, Volume *125*

# Supplemental information

# Synapsin shields a lipid monolayer from interactions with vesicles and associated structural rearrangements

**Hendrik Bruns, Titus Czajka, Charlotte Neuhaus, Maciej Jankowski, Diego Pontoni, Christian Hoffmann, Dragomir Milovanovic, and Tim Salditt**

Manuscript submitted to **Biophysical** *Journal*

**Supporting Material**

# Synapsin shields a lipid monolayer which is contact with synaptic vesicles or small lipid vesicles from structural rearrangement

Hendrik Bruns[1,‖], Titus Czajka[1,‖], Charlotte Neuhaus[1], Maciej Jankowski[3], Diego Pontoni[3], Christian Hoffmann[2], Dragomir Milovanovic[2], and Tim Salditt[1,*]

[1]Institute for X-ray physics, Friedrich-Hund-Platz 1, Göttingen, 37077, Germany
[2]Laboratory of Molecular Neuroscience, German Center for Neurodegenerative Diseases (DZNE), 10117 Berlin, Germany
[3] ESRF - European Synchrotron Radiation Facility, 71 avenue des Martyrs, 3800 Grenoble, France
[‖]These authors contributed equally
[*]Correspondence: tsaldit@gwdg.de

## A GRAZING INCIDENCE DIFFRACTION: LATTICE PARAMETERS AND PEAK FITTING

The GID data presented in Fig. 2,3(e) is the result of fitting the $\{11\}$ and $\{02\}$ peaks in the 2d plane shown in Fig. S1. The two-dimensional crystalline packing of the lipid chains is described by the lattice vectors $a$ and $b$ of a rectangular lattice. The GID peaks with inplane component of the scattering vector $q_{hk}$ (Miller indices) are related to the lattice vectors as (1)

$$q_{hk} = \frac{1}{d_{hk}} = \frac{\sqrt{h^2b^2 + k^2a^2}}{ab}\,. \tag{1}$$

From $q_{02}$, we can directly infer the lattice parameter $b$

$$q_{02} = \frac{1}{d_{02}} = \frac{\sqrt{0^2b^2 + 2^2a^2}}{ab} = \frac{2}{b} \quad \Rightarrow \quad b = 2d_{02}\,. \tag{2}$$

This relation for $b$ can then be inserted in the expression for $q_{11}$ in Eq.(1)

$$q_{11} = \frac{1}{d_{11}} = \frac{\sqrt{1^2b^2 + 1^2a^2}}{ab} \tag{3}$$

$$\Rightarrow \quad d_{11} = \frac{ab}{\sqrt{b^2 + a^2}} \overset{(2)}{=} \frac{a2d_{02}}{\sqrt{(2d_{02})^2 + a^2}} = \frac{2d_{02}}{\sqrt{\left(\frac{2d_{02}}{a}\right)^2 + 1}} \tag{4}$$

$$\Rightarrow \quad \sqrt{\left(\frac{2d_{02}}{a}\right)^2 + 1} = \frac{2d_{02}}{d_{11}} \quad \Rightarrow \quad \frac{(2d_{02})^2}{a^2} = \frac{(2d_{02})^2}{d_{11}^2} - 1 \quad \Rightarrow \quad a^2 = \frac{(2d_{02})^2}{\frac{(2d_{02})^2}{d_{11}^2} - 1} \tag{5}$$

$$\Rightarrow \quad a = \frac{2d_{02}}{\sqrt{\frac{(2d_{02})^2}{d_{11}^2} - 1}} = \frac{1}{\sqrt{\frac{1}{d_{11}^2} - \frac{1}{(2d_{02})^2}}}\,. \tag{6}$$

From the lattice vectors $a$ and $b$, one obtains the area per chain $A$ and tilt angle $\tau$, presented in Fig. 2,3,5,6. The corresponding 2d measurements in the $q_{x,y}$ and $q_z$ plane are shown in Fig.S1, S2, S3. The dark lines indicate the fit results for the three relevant prak positions $q_{xy,11}$, $q_{z,11}$, and $q_{xy,02}$. The gray lines are added as a visual reference. The peak center is determined by projecting the intensity onto the respective axis and subsequently fitted to a Gaussian. The width is used to determine the error.

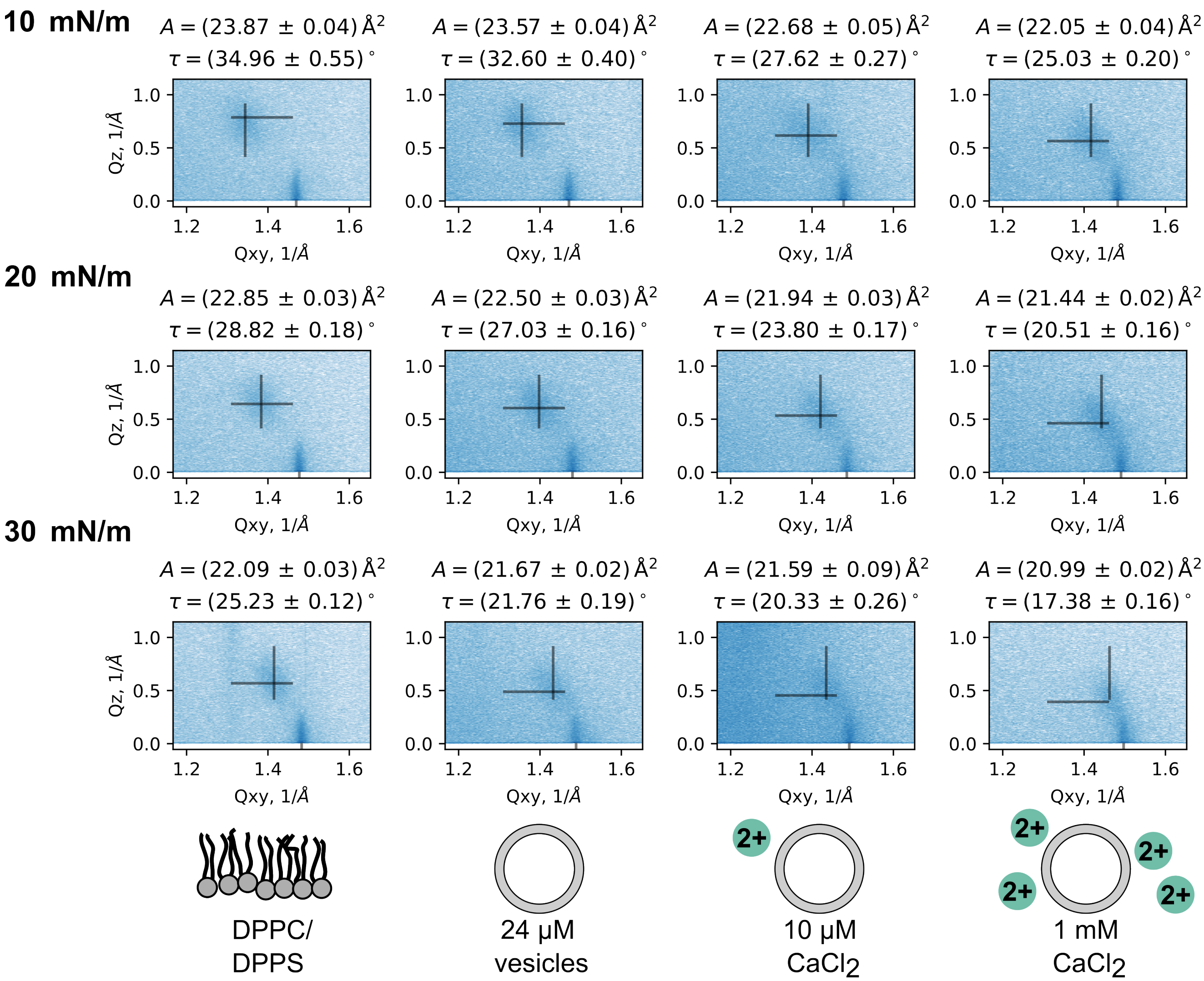


Figure S1: GID data collected at a constant pressure $p$, by scanning the linear 1d detector along angle $2\theta$. The linear 1d detector is aligned along the interface normal. Two peaks are observed; the gray lines indicate the fitted peak positions. From the peak positions ( $q_z^{11}$ $q_{xy}^{11}$ $q_{xy}^{02}$) the area per chain $A$ and the tilt angle $\tau$ is computed (see subpanel titles). The data corresponds to the $A$ results presented in Fig.3(e).

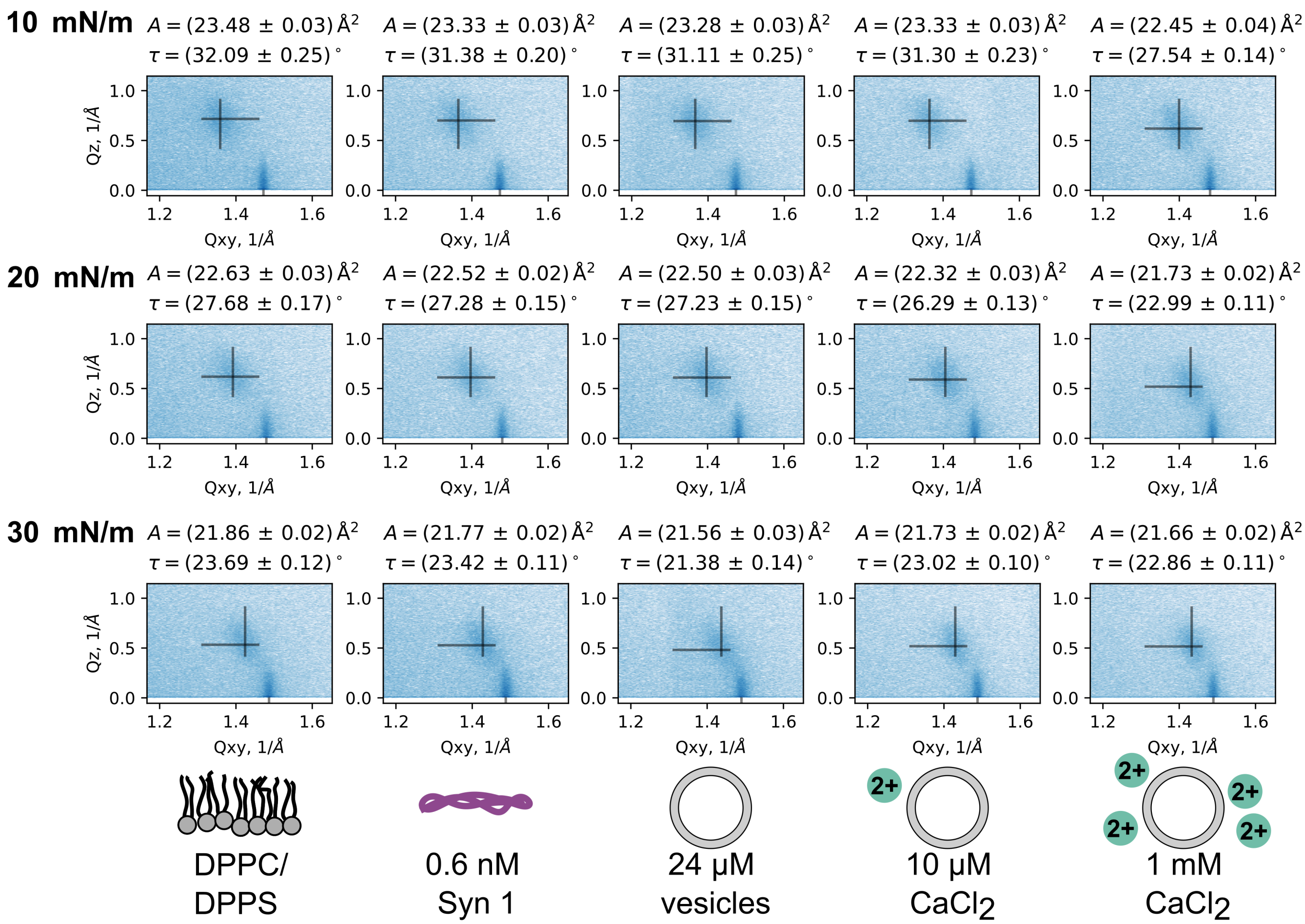


Figure S2: GID data corresponding to $A$ values presented in Fig.2(e); else same as in Fig. above.

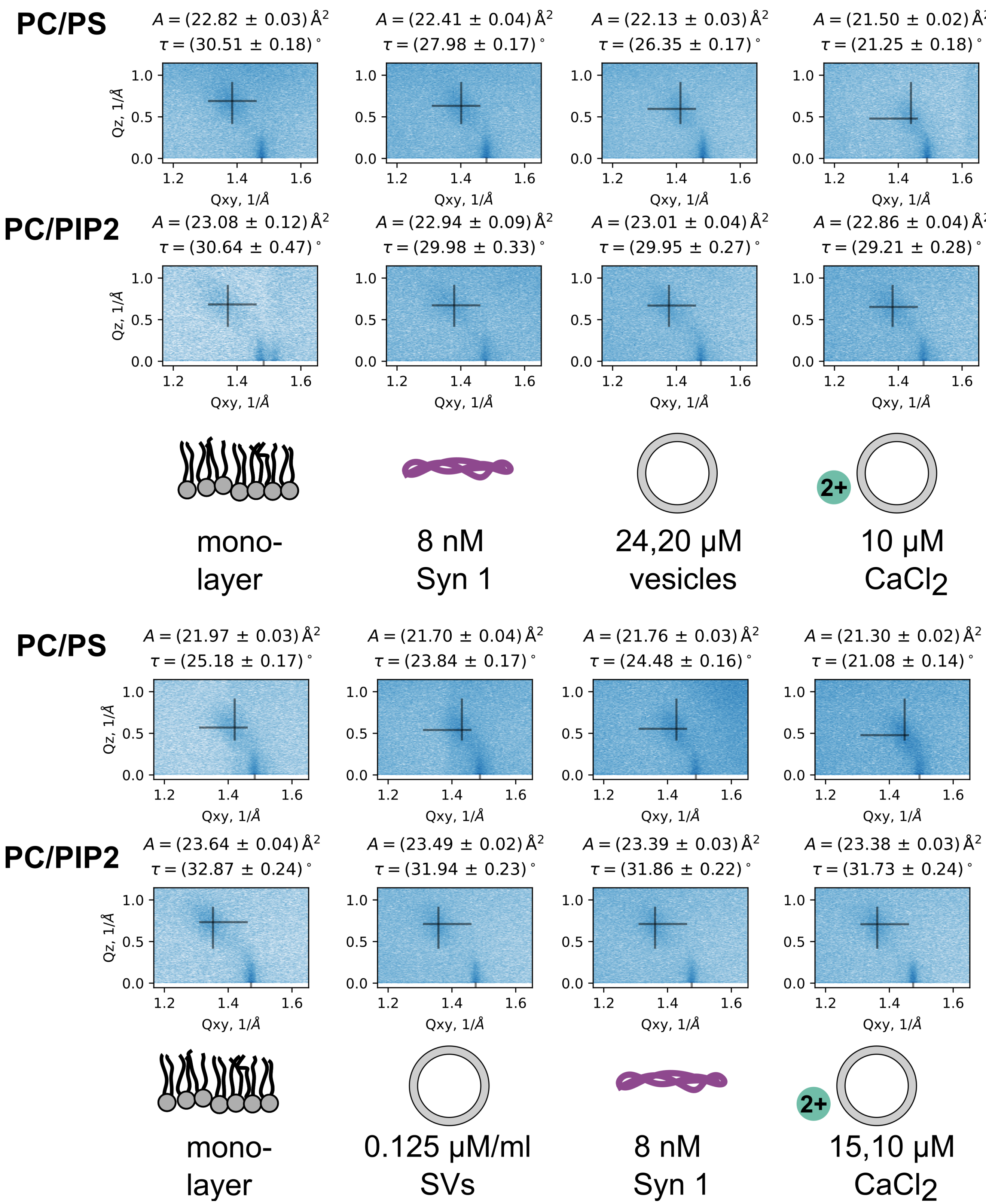


Figure S3: GID data from the small trough data, at constant area and approximate monolayer pressure of 20 mN/m. The 2d plots correspond to the data presented in Fig. 5,6.

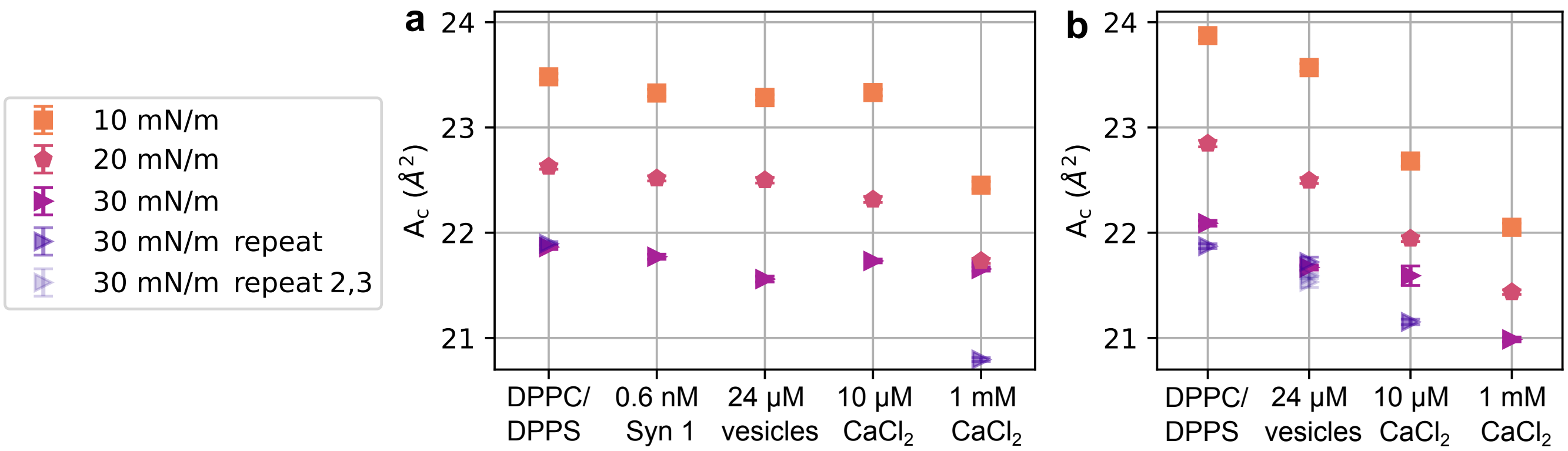


Figure S4: GID data obtained on the large trough, as presented in Fig. 2,3(e). Repeat measurements are shown at constant monolayer pressure of 30 mN/m. The 2d plots correspond to the data presented in Fig.S1 and S2

**Repredmeasurementsucibility** In order to check for reproducibility of the states of the individual monolayers in Fig. 2,3(e) repeated measurements where performed at a surface pressure 30 mN/m and are shown in purple in Fig. S4. The monolayer repeat measurements are acquired after going to the lower pressures showing a similar area per chain $A_c$. Fig. S4(b) shows three repeated measurements at 30 mN/m, following the acquisition of the two lower pressures. These three measurements show a slow decay of $A_c$ which can be attributed to the reordering of lipids finding the optimal packing at this pressure. This could also explain the greater variations in $A_c$ at 30 mN/m as the injection process varied in duration. Accordingly, the lipids were kept at 30 mN/m for a varying duration, while the measurement script for the lower film pressures consistently adjusted the trought to the desired pressure, and then waited for 5 min before starting the measurement. In addition, we note that the initial measurement of the 30 mN/m monolayer after $CaCl_2$ injection deviates from the repeated measurement (see Fig. S4 10 mM $CaCl_2$ (a) and 10 $\mu$M $CaCl_2$ (b)). This is indicative of either a delayed interaction or a required lower membrane surface pressure $\Pi$ for the interaction.

## B X-RAY REFLECTIVITY ANALYSIS

Since the XRR fits had a tendency towards overfitting with a three-box model, the modelling of a separate protein layer had to be given up. It was found, that the information that distinguished different sets of parameters from each other was contained most heavily at $q > 0.6\,\text{nm}^{-1}$, where the X-ray data was too close to the noise level to yield definitive results. A particular property of the three-box models was that they required the space between monolayer and subphase to have a density below that of the subphase, which was deemed to be an unphysical solution.

To reduce the complexity of the model, given the low information content available for higher spacial frequencies (high $q$), a two box model was chosen (as illustrated in Fig. S6). This reduced the number of parameters and thus led to less variation in the fit results for different sets of initial parameters, but still did not yield a single best fit result. To achieve this, we further constrained the model by setting the the interfacial width $\sigma$ equal for all three interfaces. Furthermore, we set an upper limit for the tail density $\rho_t < (0.93 \cdot \rho_s)$.

The fitting procedure itself comprised a least-squares minimisation algorithm which was fed different sets of parameters. As criterion for the goodness of fit, the adjusted coefficient of determination $R^2_{adj}$ was used. To test the validity of the calculated best least-squares fit from a certain set of input parameters, we chose different parameters based on the concept of a Metropolis algorithm: The set of parameters that is an input to each fit is taken as the initial probability distribution and the resulting value of $R^2_{adj}$ corresponds to the energy of the system in the original algorithm. The algorithm then iterates through multiple sets of parameters by proposing a new parameter configuration based on the old configuration, but now each individual parameter is shifted by an amount $\pm\Delta p_i < p_i$ chosen from a normal distribution centred around $p_i$. This new set of parameters is accepted when $(R_i/R_{i+1})^n > U[0;1]$, where $U[0;1]$ denotes a uniform random number in the interval between 0 and 1. The goal of the algorithm is to obtain a large variety of different fits that are on the other hand not sampling too many configurations where the least-squares fit algorithm fails. We obtained good results by choosing $n = 4$ and $\Delta p_i = \mathcal{N}(p_i, \Delta * p_i)$ where $\Delta$ was varied from 0.5 to 0.002.

Even though the least-squares minimisation is not a Markovian process, and the Metropolis algorithm is hence not strictly applicable here, we nonetheless found it to be a reasonable way to generate a multitude of different fit parameters and output configurations. The different configurations then allowed us to assess whether the models yielded different fit results of similar

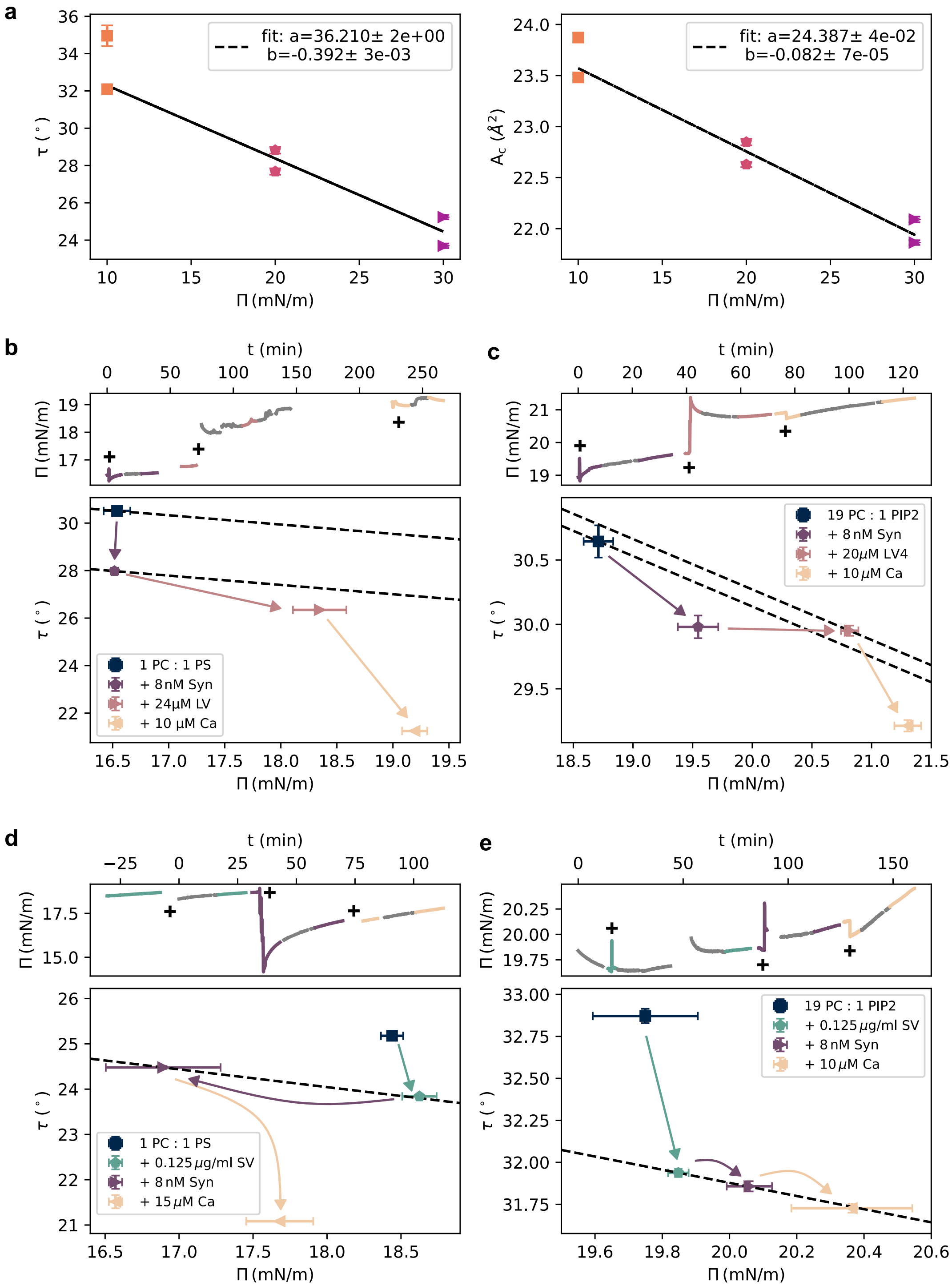


Figure S5: (a) Monolayer pressure dependence of the chain tilt angle $\tau$ and area per chain $A_c$, along with linear fits. These fits are then used as reference isotherms in (b-e) ($\tau$) and in Fig. 5,6 ($A_c$). (b-e) Tilt angle $\tau$ in response to injections of Syn1, lipid vesicle, synaptic vesicles and $CaCl_2$, corresponding to Fig. 5,6 (main manuscript). The response in $\tau$ is similar to $A_c$.

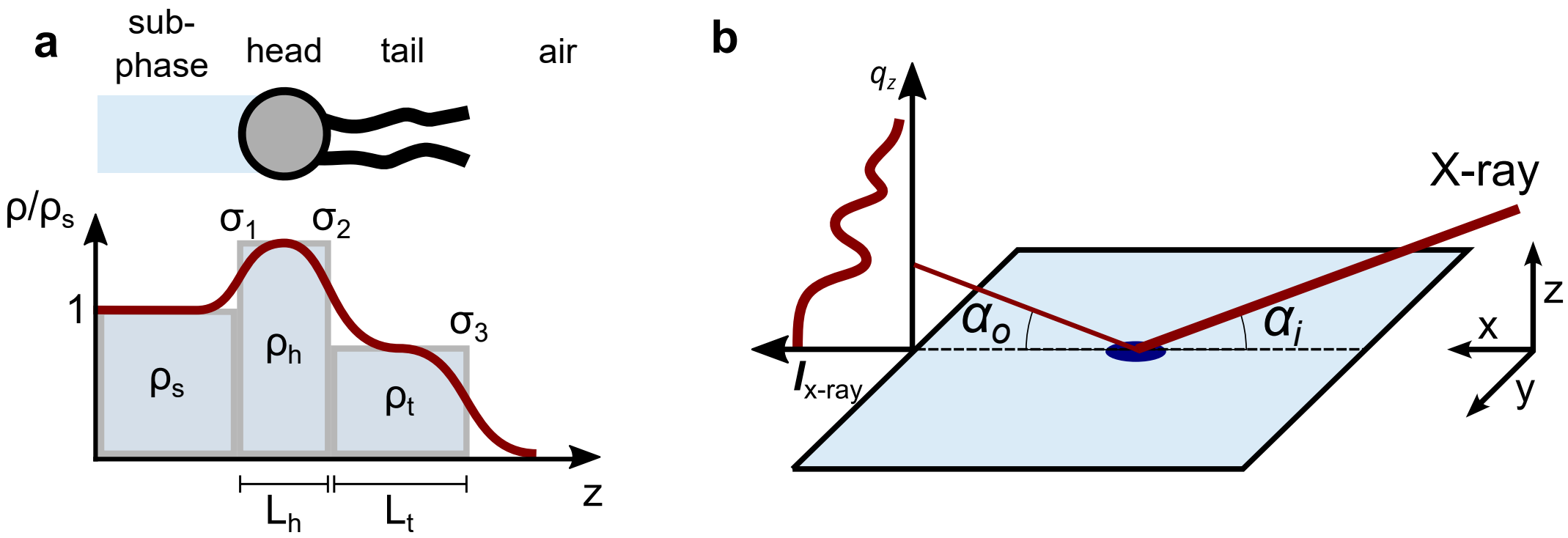


Figure S6: (a) XRR two box density model for the lipid head and tail group, illustrating the parameterisation of the density profile $\rho$. These parameters are varied to fit the reflectivity curves recorded as sketched in (b).

$R^2_{adj}$ or rather a single global optimum.

## REFERENCES

1. Marsh, D., 2012. Lateral order in gel, subgel and crystalline phases of lipid membranes: Wide-angle X-ray scattering. *Chemistry and physics of lipids* 165:59–76.
